# Chainmail catalysis for advanced water treatment: overcoming the stability–reactivity tradeoff in Fenton-like oxidation of micropollutants

**DOI:** 10.1186/s40580-025-00526-w

**Published:** 2025-12-19

**Authors:** Quang Viet Ly, Lele Cui, Yuri Park, Long D. Nghiem, Hanh Tien Nguyen, Van-Duong Dao, Soryong Chae, Yuhoon Hwang

**Affiliations:** 1 Smart Green Transformation Center (GREEN-X), VinUniversity, Vinhomes Ocean Park, Gia Lam, Hanoi, 100000 Vietnam; 2 Institute of Environmental Technology, Seoul National University of Science and Technology, Seoul, 01811 Republic of Korea; 3 Department of Mechanical Engineering, The Hong Kong Polytechnic University, Kowloon, 999077 Hong Kong SAR China; 4 Centre for Technology in Water and Wastewater, University of Technology Sydney, Ultimo, NSW 2007 Australia; 5 Faculty of Biotechnology, Chemistry and Environmental Engineering, PHENIKAA University, Hanoi, 100000 Vietnam; 6 Department of Chemical and Environmental Engineering, University of Cincinnati, 2901 Woodside Drive, Cincinnati, OH 45221–0012 USA; 7 Department of Environmental Engineering, Seoul National University of Science and Technology, Seoul, 01811 Republic of Korea

**Keywords:** Chainmail catalysis, Ultrathin carbon layer, Advanced oxidation processes, Structural parameters, Micropollutants

## Abstract

Recent developments in chainmail catalysis present a promising solution to the longstanding challenge of catalytic deactivation in traditional advanced oxidation processes (AOPs) for water reuse. This strategy involves encapsulating nanocatalysts within protective carbon shells, which significantly improves their stability and enhances the degradation kinetics of micropollutants. The unique architecture of these materials promotes efficient electron transfer between the transition metal core and the surrounding carbon matrix, giving rise to a range of intriguing and unprecedented phenomena. Given its strong potential, this review provides a comprehensive overview of the latest developments in chainmail catalysis, highlighting its advantages over conventional AOPs. This review critically examines experimental results, theoretical insights, and conflicting observations related to the generation of reactive species and degradation mechanisms of micropollutants. The influence of key parameters (such as catalyst structure and activity, carbon shell morphology and thickness, heteroatom doping, surface functionalization, and the composition and redox state of the metal core) on chainmail catalytic performance is also thoroughly analyzed. Furthermore, the review assesses the practical viability of chainmail catalysis in real-world water treatment applications by examining the impact of operating conditions and water matrices on chainmail catalytic performance. Finally, this review outlines key technological challenges and proposes a roadmap to guide future research and the practical implementation of chainmail catalysis for efficient removal of micropollutants.

**Graphical abstract:**

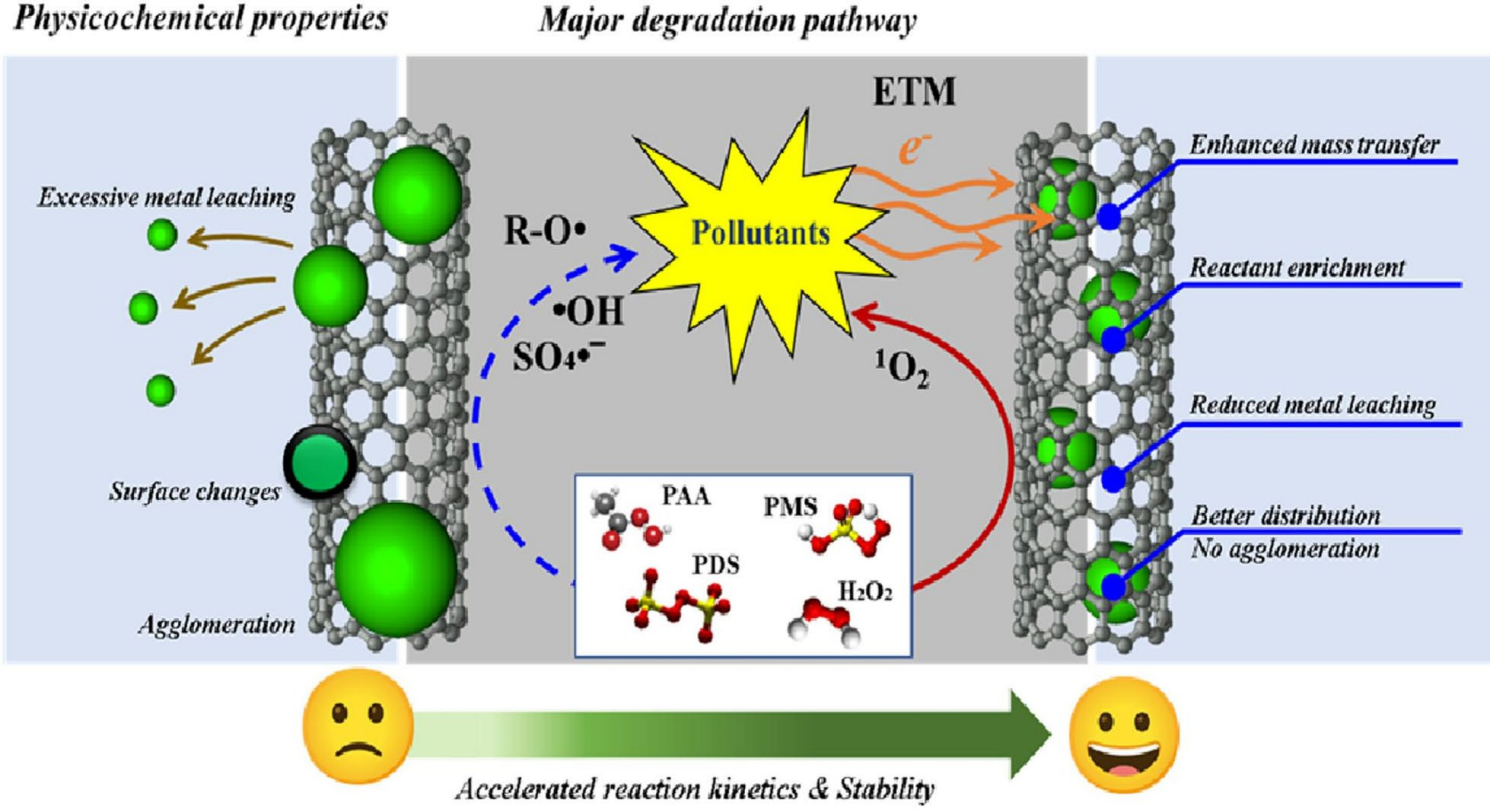

## Introduction

Rapid urbanization, population growth, and economic development have contributed to a global decline in freshwater availability [1]. As of 2022, approximately one-quarter of the global population experienced severe water shortages, and freshwater demand is expected to exceed supply by 40% by 2030 [2, 3]. To address this growing challenge, the reuse and recycling of water have been strongly promoted under the United Nations Sustainable Development Goal 6 [4].

However, the practical implementation of water reuse faces significant challenges, primarily due to the widespread presence of refractory organic micropollutants such as pesticides, endocrine-disrupting compounds (EDCs), and pharmaceutical and personal care product (PPCP) residues [5]. For example, in South Korea, at least 29 pharmaceutical and antimicrobial compounds have been detected in major river systems [6, 7]. In China, 92 PPCPs and 36 EDCs have been frequently found in surface waters, with compounds such as 4-nonylphenol, carbamazepine, oxytetracycline (OTC), ofloxacin (OFX), sulfamethoxazole (SMX), and tetracycline (TC) commonly occurring at concentrations of several μg/L [8]. These contaminants are chemically stable due to their complex molecular structures, small size, and low biodegradability, posing potential toxicological risks to both aquatic ecosystems and human health [9, 10]. For example, SMX has been reported to alter the developmental morphology of *Caenorhabditis elegans* [11]. Triazine herbicides can disrupt photosynthesis and carbon metabolism in the phytoplankton *Phaeodactylum tricornutum* [12, 13]. Bisphenol A (BPA) is known to interfere with reproductive and developmental systems and has been linked to metabolic disorders [14]. Of particular concern is the connection between environmental contamination and antimicrobial resistance (AMR), which is projected to become the leading cause of global mortality by 2050; in 2019 alone, AMR was associated with 4.95 million deaths [15, 16]. Consequently, the effective removal of these micropollutants is critical to ensuring the safety and long-term viability of water reuse systems.

Among the various treatment strategies available, advanced oxidation processes (AOPs) have emerged as powerful and versatile solutions due to their proven effectiveness in degrading a wide range of micropollutants. The performance of AOPs primarily relies on the generation of hydroxyl radicals (•OH) and sulfate radicals (SO_4_•^−^), which exhibit the highest redox potentials among reactive oxygen species (ROS) i.e., *E*^0^(•OH/OH‾) = 1.9–2.7 V versus NHE, and *E*^0^(SO_4_•^−^/SO_4_^2^‾) = 2.6–3.1 V versus NHE. [17, 18]. These strong oxidants are capable of decomposing even the most recalcitrant pollutants, often leaving no residual organic compounds [19, 20]. However, due to their short lifespans, ROS must be generated in situ by activating stable oxidants, such as hydrogen peroxide (H_2_O_2_), peroxymonosulfate (PMS), persulfate (PS), and peracetic acid (PAA), through external energy sources (e.g., ultraviolet (UV) light, ultrasound, or heat) or by electron transfer involving transition metals (TMs), transition metal oxides (TMOs), or carbon-based materials [21, 22]. To lower energy consumption and enhance cost-effectiveness, electron transfer-based catalytic AOPs have been extensively studied for their practical potential in real-world applications [23].

Among these processes, heterogeneous AOPs that utilize solid TM/TMO catalysts are particularly appealing due to several key advantages, including enhanced adaptability to varying environmental conditions, enhanced catalyst reusability, and better suitability for small-scale, decentralized modular systems [23, 24]. However, a major obstacle to their widespread adoption is the reduced stability of catalysts during long-term operation [10, 25].

To address this challenge, the concept of chainmail catalysis has emerged as a promising solution. In this approach, metal active centers are encapsulated within ultrathin graphitic carbon layers [26]. This unique structural configuration significantly improves the durability of metal catalysts, especially under harsh conditions such as strong acidity or alkalinity, elevated temperatures, or highly oxidative environments. The graphitic shell serves as a protective barrier, shielding the metal active sites from direct exposure to the reaction medium while maintaining high catalytic performance [27].

In chainmail catalysis, catalytic activity is driven by electron transfer between the encapsulated metal core and the surrounding carbon shell [25, 27]. This electron penetration mechanism influences the generation of ROS and alters the degradation pathways of micropollutants compared to conventional methods, offering new insights into the underlying thermodynamics and kinetics of these reactions. For example, the degradation of BPA using Co_3_O_4_ confined within carbon nanotubes (Co_3_O_4_@CNTs) to activate PAA achieved a rate constant of 0.188 min^−1^, nearly four times higher than that of conventional Co_3_O_4_ loaded on the exterior of CNTs (Co_3_O_4_/CNTs), which had a rate constant of 0.049 min^−1^. The confined catalyst also demonstrated excellent durability, maintaining its performance over 10 consecutive cycles. In this system, ^1^O_2_ and O_2_•‾ were identified as the dominant ROS, whereas alkyl radicals such as CH_3_C(O)O• and CH_3_C(O)OO• were more prevalent in conventional AOP systems [28]. Similar results have been observed in other chainmail configurations. For example, carbon-coated Fe_3_O_4_ (Fe_3_O_4_@C) activated H_2_O_2_ and consistently removed approximately 80% of phenol across 14 operational cycles [29]. Other notable systems include N-doped carbon nanotube-confined Fe/Mn nanoparticles (FeMn@NCNT) for PMS activation [30], oxidized CNT-encapsulated Fe/Co Prussian blue analogues (Fe/Co PBA@OCNTs) with H_2_O_2_ [31], Fe_3_O_4_@C/PS under visible light [32], Co@CNT/PMS [33], CuFe_2_O_4_@C/PS [34], FeOCl confined reduced graphene oxide on carbon cloth (FeOCl/CC@rGO) [25], and FeOCl confined Metal–organic framework (FeOCl@MOF) [35].

Despite promising results reported in the literature, the industrial-scale application of chainmail catalysis in heterogeneous AOPs remains largely unexplored, primarily due to the absence of a well-defined conceptual framework and limited understanding of the underlying mechanisms. One key uncertainty lies in determining the optimal thickness of the carbon encapsulation layer. For example, Deng et al. [26] proposed that the chainmail structure is only effective when the carbon layer is fewer than three layers thick, approximately a few nanometers, to ensure efficient electron transfer [26]. While this criterion has been widely accepted for the effective removal of organic contaminants [36–39], several studies have demonstrated successful degradation even when the carbon layer is considerably thicker (up to approximately 100 nm) [28, 35, 40]. Based on the current body of research, this work considers chainmail catalysis as those in which the encapsulating carbon layer is less than 100 nm thick, and regards such materials as relevant to the evaluation of chainmail catalysis for micropollutant removal in water treatment applications.

Several review efforts have explored this emerging area of research. For example, Zhao et al. provided a general overview of chainmail catalysis for environmental remediation, though their focus was primarily on the removal of gaseous pollutants such as NH_3_, NO_x_, and volatile organic compounds [41]. More recent reviews have centered on synthetic strategies for developing encapsulated catalysts [42]. Yan et al. [43] conducted a comprehensive review of metal–carbon hybrid materials for PS activation. However, it is important to note that these metal–carbon hybrids still allow full exposure between the metal catalysts and reactants. This contrasts with chainmail catalysts, which are designed to minimize direct contact between the inner metal core and external oxidants.

To the best of our knowledge, this work represents the first comprehensive analysis of chainmail catalysis specifically for the degradation of micropollutants, to elucidate key mechanistic insights and evaluate practical implications. Specifically, this review aims to unveil several fundamental questions, including: What are the primary mechanisms governing micropollutant removal? Which types of ROS are involved? How do electron transfer dynamics and degradation thermodynamics differ in chainmail systems relative to conventional AOPs? How do the physicochemical properties of hybrid materials, operational parameters, and water matrix components influence the performance of chainmail-based AOPs compared to conventional approaches?

For consistency and clarity throughout this review, we define catalysts encapsulated within a carbon shell as TM@C, representing chainmail catalysis, and catalysts supported on carbon materials as TM/C, representing conventional systems. In summary, this critical review aims to address key knowledge gaps by focusing on four key areas: (1) elucidating the distinct characteristics and underlying mechanisms of chainmail catalysis in comparison to conventional AOPs; (2) evaluating the influence of the physicochemical properties of chainmail catalysts; (3) examining the effects of operational parameters and water matrix components on the degradation of emerging micropollutants; and (4) outlining a roadmap to guide future research and practical developments in this field.

## Distinctive advantages of chainmail catalysis over conventional AOPs

### Enhanced stability of carbon-encapsulated nanocatalysts

**Fig. 1:**
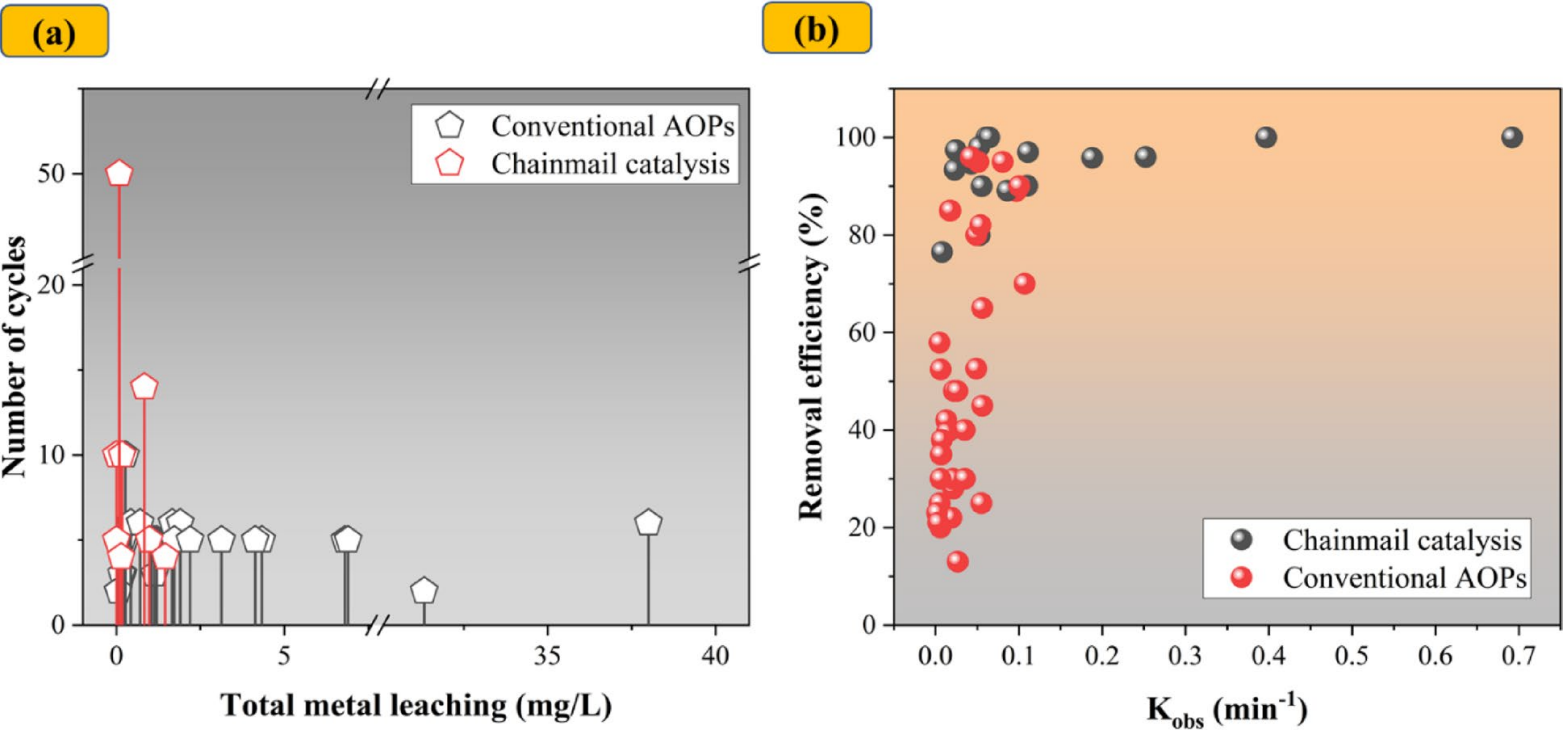
Comparative analysis of traditional catalytic degradation and chainmail catalysis systems. **a** Number of operational cycles versus total metal leaching (mg/L) **b** Removal efficiency (%) and corresponding degradation rate constants (k_obs_, min^−1^). Data was collected from previous works [25, 28, 29, 34, 35, 44, 51, 53, 65, 68–85]

The distinctive properties of chainmail catalysis over conventional catalysis have been summarized in Table 1. There is increasing consensus in the literature that catalytic processes based on the chainmail concept offer superior stability compared to conventional approaches (Fig. 1). For example, electro-Fenton (EF) systems utilizing FeCo@C electrodes demonstrated outstanding performance, maintaining a ciprofloxacin (CIP) removal efficiency of 95.5% after 50 operational cycles—only a minor decrease from the initial 99.8% removal rate [44]. In another study, Liu et al. compared two systems of Co_3_O_4_@CNTs and Co_3_O_4_/CNTs for PAA activation in BPA degradation. After 10 consecutive cycles, the confined catalyst retained 95% degradation efficiency, whereas the unconfined counterpart achieved only 7% [28].

**Table 1:** Key differences between chainmail catalysts and conventional heterogeneous AOP catalysts

Criteria	Chainmail catalysts (TM@C)	Conventional catalysts (TM/C, TM/TMO)
Structure architecture	Metal core fully encapsulated by ultrathin (1–100 nm) graphitic carbon shell	Bare TM/TMO or TM/TMO integrated externally on a carbon template
	Strong metal–carbon interfacial bonding and confined architecture	Active sites directly exposed to solution
Synthesis	Requires precise control of shell thickness	No specific requirement
	Complexity in fabrication to generate nanoconfinement and interfacial interactions	Typically simpler synthesis
Stability and metal leakage	Prevent agglomeration, less leaching	High agglomeration, susceptible to leaching
	Maintain structural integrity after repeated cycles	Gradual loss of crystallinity (XRD) and active sites (XPS)
Electrochemical properties	Carbon shell shifts the Fermi level, improving conductivity	Conductivity depends on the intrinsic properties of TM/TMO
	Lower charge transfer resistance, as evidenced by EIS,	Could be higher charge-transfer resistance, and lower electron transfer
	Higher reactivity, as evidenced by CV or LSV	
Mass transfer & adsorption	Enhanced π–π interactions and hydrogen bonding from graphitic layers	Adsorption is dominated by surface functional groups of the support
	Confined geometry improves local concentration and interfacial reactions	
	→ Stronger adsorption of aromatic micropollutants	→ Lower mass transfer efficiency of micropollutants
Catalytic activation	Dual activation pathways	Primarily governed by the radical pathway
	Possible involvement of radicals •OH or •SO_4_‾	•OH or •SO_4_‾generated by direct reactant activation by the metal redox cycle
	Enhanced non-radical pathways (^1^O_2_ dominant)	Possible involvement of ^1^O_2_
	Electron-transfer mediated (ETM) via conductive carbon shell as an electron shuttle	Limited ETM contribution
Degradation performance & kinetics	Could be more selective to some micropollutants	Have a broader range of micropollutant degradation
	Higher kinetic (kobs)	Could be a slower kinetic

More importantly, the distinct carbon encapsulation architecture renders these catalysts more resistant to environmental degradation over extended periods. For example, polydopamine (PDA) effectively stabilized CuFe_2_O_4_ in aqueous solution for over one month, maintaining structural integrity [40]. Similarly, nanoscale zero-valent iron (nZVI) encapsulated within a mesoporous carbon shell preserved its morphology and crystallinity for up to 20 days [45]. In another study, Co_3_O_4_ confined within nitrogen-doped carbon (Co_3_O_4_@NC) retained its strong magnetic properties even after exposure to aqua regia followed by hydrothermal treatment [46]. These stability-enhancing characteristics offer significant advantages in terms of catalyst handling, storage, and transport, thereby improving their feasibility for real-world applications.

Several factors contribute to the enhanced stability of carbon-confined catalysts. The carbon encapsulation acts as a protective barrier, shielding the core metal from direct environmental exposure. This carbon layer significantly reduces metal leaching under acidic conditions and inhibits the binding of OH⁻ ions to metal-active sites in neutral or alkaline environments [26]. For example, a threefold reduction in iron leaching (from 2.8 to 0.8 mg/L) was observed when carbon-coated catalysts were used [29]. Similarly, the introduction of an ultrathin graphitic layer significantly suppressed iron leaching from 31.32 to 0.18 mg/L in bare FeOCl/CC compared to FeOCl/CC@rGO [25], and from 14.7 to 0.58 mg/L in bare Fe_x_P/C compared to Fe_x_P/C@rGO [47], a trend consistent with previous studies [36]. These results were further supported by other analyses. X-Ray Photoelectron Spectroscopy (XPS) analyses revealed minimal changes in metal composition following multiple operational cycles [48]. Using electrochemical analyses, Li et al. (2018) observed the absence of redox peaks associated with the Fe^3+^/Fe^2+^ and Co^3+^/Co^2+^ cycles in FeCo@NC after acid treatment, highlighting the strong protective effect of the graphene shell [49]. This controlled ion leaching behavior is likely attributed to the formation of robust metal–carbon interfacial bonds formed at the junction between the metal terminations and the carbon lattice [50].

Beyond reducing metal leaching, carbon materials also enhance catalytic performance by promoting efficient metal redox cycling [10, 25, 29, 51]. Cui et al. [25] reported that FeOCl/CC@rGO exhibited a tenfold higher proportion of Fe^2+^ in the total iron leached (33.3%) compared to FeOCl/CC (2.1%). This improvement was attributed to the high electrical conductivity of the rGO shell, which facilitated the one-electron reduction of Fe^3+^ to Fe^2+^ [25]. From an environmental perspective, controlling the release of toxic metal ions such as Co, Fe, and Mn is crucial for complying with wastewater discharge regulations. The carbon layer also helps prevent the formation of complexes between organic degradation intermediates and catalytic sites, thereby preserving catalytic activity [46, 52]. Notably, catalytic performance can often be restored through simple thermal treatment or solvent washing, which effectively removes adsorbed organic impurities and extends the catalyst lifespan [29, 30, 38, 53–55]. For example, the reactivity of Fe_2_O_3_@CNT was fully recovered after ethanol washing following five consecutive operational cycles [56].

**Fig. 2:**
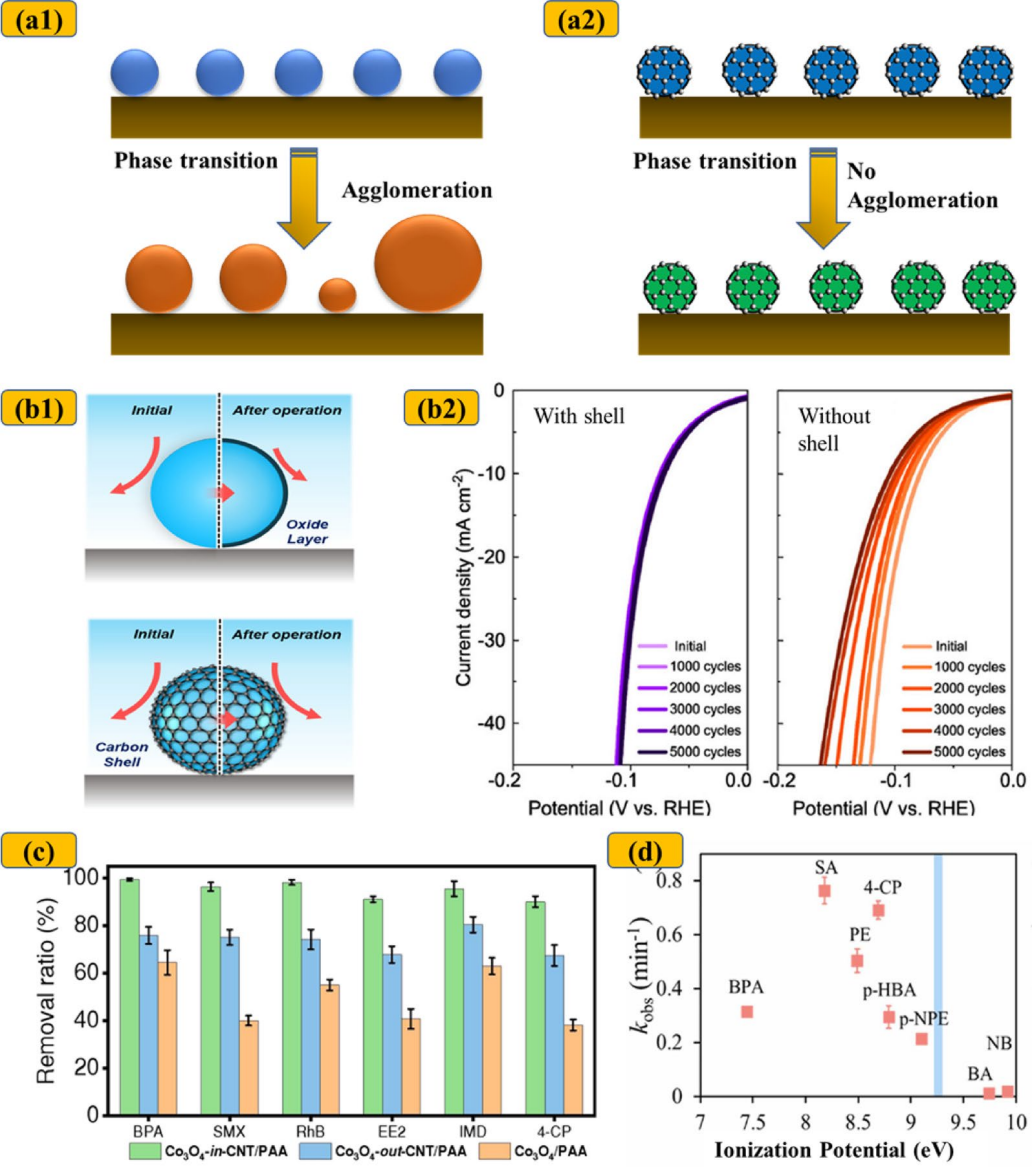
**a** Schematic illustration of particle agglomeration during long-term electrocatalysis between bare catalysts (**a1**-left) versus carbon-confined catalysts (**a2**-right). **b1** Schematic illustration of surface change between bare nanocatalysts (top) versus carbon-confined catalysts (bottom). Adapted with permission from [86]. Copyright (2022) American Chemical Society, and **b2** Electrochemical stability of FeP catalysts over 5000 cycles experiments with and without carbon shell. Reprinted with permission from [87]. Copyright (2017) American Chemical Society. **c** Comparison of BPA, SMZ, RhB, EE2, IMD, and 4-CP removal using Co_3_O_4_@CNT, Co_3_O_4_/CNT, and Co_3_O_4_ for PAA activation. Reproduced from [28], licensed under CC BY 4.0. **d** Degradation of various organic micropollutants using the 5-FeMn@NCNT/PMS system. Reproduced from [30], with permission from Elsevier

Reducing TM particles to the nanoscale often results in severe agglomeration, primarily due to their high surface energy and magnetic dipole interactions (Fig. 2) [57–59]. Over time, thermodynamically driven agglomeration further reduces catalyst surface area and reduces overall efficiency [60]. Consequently, nanocatalysts may be unable to fully utilize their inherently high surface area in AOPs [61]. Carbon encapsulation offers an effective solution by providing spatial confinement that prevents particle aggregation, thereby maintaining high surface area and catalytic activity [51, 61–64]. For example, Li et al. reported the uniform dispersion of FeCo bimetallic nanoparticles (25–60 nm) within a carbon shell [65], a finding corroborated by several other studies [66, 67].

### Removal efficiency and degradation kinetics of chainmail catalysts

Recent studies have demonstrated that chainmail catalysis can synergistically combine the advantages of both metallic and non-metallic components, often outperforming conventional AOPs in contaminant degradation (Fig. 1). For example, Yang et al. (2019) reported complete removal of methylene blue (MB) within one hour using Fe_2_O_3_@CNT, significantly exceeding the degradation efficiencies observed with Fe_2_O_3_/CNT (30%), CNT (50%), and bare Fe_2_O_3_ (15%) in the presence of H_2_O_2_ [56]. Similarly, CuFe_2_O_4_@PDA/H_2_O_2_ achieved over 99% MB degradation in 30 min, approximately double the efficiency of unmodified CuFe_2_O_4_/H_2_O_2_ [40]. Encapsulating FeOCl within a MOF layer also led to superior performance, with over 90% BPA degradation compared to approximately 80% with bare FeOCl and only 40% with the MOF alone [35]. In another study, Co@C exhibited significantly higher reactivity for PMS activation than either soluble Co^2+^ or unprotected Co_3_O_4_, achieving 100%, 71.8%, and 14.8% phenol degradation, respectively, within 10 min. Notably, the carbon shell alone, following the removal of the core metal catalyst, showed negligible catalytic activity (< 10%) for phenol degradation [50], highlighting the critical role of synergistic interactions in the effectiveness of chainmail catalysts.

Enhanced degradation kinetics have also been consistently observed with chainmail catalysts. For example, the observed rate constant (k_obs_) for sulfamethazine (SMT) degradation using Fe^0^@CNTs was 0.061 min^−1^, which is 2.7, 3.4, and 9.8 times higher than those obtained with Fe_3_O_4_/CNTs, Fe_3_O_4_/Fe^0^/CNTs, and CNTs alone, respectively [88]. Similarly, FeMn@NCNT demonstrated superior PMS activation for the rapid degradation of acetamiprid, outperforming MnC, FeC, Fe_3_O_4,_ and MnFeO catalysts [39]. In an EF system, the use of a Fe^0^@CNT cathode yielded a phenol degradation rate of 0.199 min^−1^, nearly 10 times higher than that achieved by Fe^0^/CNT (0.02 min^−1^) [89]. Furthermore, Xiao et al. reported that a FeCo@C cathode achieved a CIP degradation rate constant of 1.44 min^−1^, representing a 7- to 144-fold increase over conventional cathodes [44].

Several studies have also demonstrated the versatility of chainmail catalysts for the degradation of various contaminants, highlighting their potential for real-world water treatment applications. For example, Qin et al. reported that a MnFe_2_O_4_@C-NH_2_/H_2_O_2_ system achieved over 95% removal of three different antibiotics, ofloxacin (OFX), amoxicillin (AMX), and tetracycline (TC), within 180 min [51]. Similarly, Co@NC, when coupled with SO_3_^2^‾ as a co-reactant, effectively degraded a range of dyes, including acid orange 7 (AO7), reactive brilliant blue (KN-R), and Methylene Blue (MB), with removal efficiencies of 92%, 96%, and 90%, respectively. In another study, the Co@NC/PMS system demonstrated high efficacy in oxidizing various dyes such as Rhodamine B, MB, Orange II, methyl violet (MV), and methyl orange (MO), achieving removal efficiencies of 84.1%, 96.0%, 100%, 97.4%, and 97.5%, respectively, within 120 min [37].

Despite the encouraging performance of chainmail catalysts, several studies have highlighted their limited efficacy against certain organic compounds, indicating selective reactivity dependent on the physicochemical characteristics of the target pollutants. For example, the Fe_2_O_3_@CNT/H_2_O_2_ system demonstrated high removal efficiencies for cationic compounds such as MV, crystal violet (CV), chrysoidine, and malachite green oxalate, but exhibited significantly lower removal efficiencies for anionic compounds such as MO, chromotrope 2R (C2R), and neutral aniline [56]. The nature of functional groups on target molecules also plays a critical role in determining degradation efficiency. For example, Mn_3_O_4_@C/PS exhibited higher reactivity toward compounds like Rhodamine B (RhB), MO, and TC, which contain electron-donating groups, compared to norfloxacin (NFX), a compound with electron-withdrawing groups [90]. Duan et al. further demonstrated that the FeMn@NCNT/PMS system was ineffective in oxidizing compounds with ionization potentials (IPs) exceeding 9.2 eV (Fig. 2) [30], suggesting that IP may serve as a predictive indicator of a compound’s susceptibility to chainmail catalysts.

Besides, the underlying degradation mechanisms could also be associated with the generation of ROS (e.g., •OH, SO_4_•‾, and ^1^O_2_). These ROS possess high electrophilic reactivity and preferentially attack electron-rich compounds or compounds bearing functional groups such as dialkylamino (–NR_2_), alkoxy (–OR), amino (–NH_2_), and hydroxyl (–OH). In contrast, molecules containing electron-withdrawing groups, such as carboxyl (–COOH) or halogens (–F, –Cl, or –Br), tend to be less reactive under ROS-driven degradation [18, 91]. Consequently, the selectivity of chainmail catalysts is fundamentally governed by the electronic structure of the target contaminants and the ROS generation pathway.

## Degradation pathways of micropollutants in chainmail-Fenton catalysis

In heterogeneous catalytic systems, the overall reaction typically involves four fundamental steps: (1) diffusion of reactant molecules to the catalyst surface, (2) adsorption onto active sites, (3) catalytic interaction leading to the generation of ROS, and (4) desorption and diffusion of the resulting products back into the bulk solution [92–94]. Chainmail catalysts, characterized by carbon-confined TM heterostructures, introduce unique physicochemical features that set them apart from conventional systems. These include improved mass transfer dynamics and distinctive electrochemical interactions at the metal–carbon interface, which collectively promote the selective generation of ROS. Such characteristics fundamentally distinguish chainmail catalysis from conventional catalytic processes.

### Thermodynamic and mass transfer kinetics

Adsorption is widely recognized as a critical preliminary step in catalytic oxidation processes, including those mediated by chainmail catalysis. Numerous studies have demonstrated a strong correlation between adsorption capacity and overall removal efficiency in these systems. For example, Chen et al. reported a significantly accelerated degradation rate of TC using NiFe_2_O_4_@C relative to unconfined NiFe_2_O_4_, which was attributed to the significantly larger specific surface area (S_BET_) and pore volume of the confined catalyst (72.23 m^2^/g and 0.17 cm^3^/g, respectively) compared to the unconfined counterpart (22.59 m^2^/g and 0.12 cm^3^/g) [95]. Similarly, Co@NG-900 achieved a k_obs_ for phenol degradation of 0.397 min^−1^, which was approximately 7.1 times higher than that of Co_x_O (k = 0.056 min^−1^); this is aligned with their respective S_BET_ values of 139.4 m^2^/g and 54.3 m^2^/g [96]. In contrast, the poor removal of MO, C2R, and neutral aniline by Fe_2_O_3_@CNT [56], as well as phenol by Mn_3_O_4_@C [61], was primarily attributed to limited adsorption affinity. The superior adsorption performance of carbon-confined catalysts over bare metal catalysts is attributed to the strong π–π interactions and hydrogen bonding between organic pollutants and the graphitic carbon layers [34, 97].

**Fig. 3:**
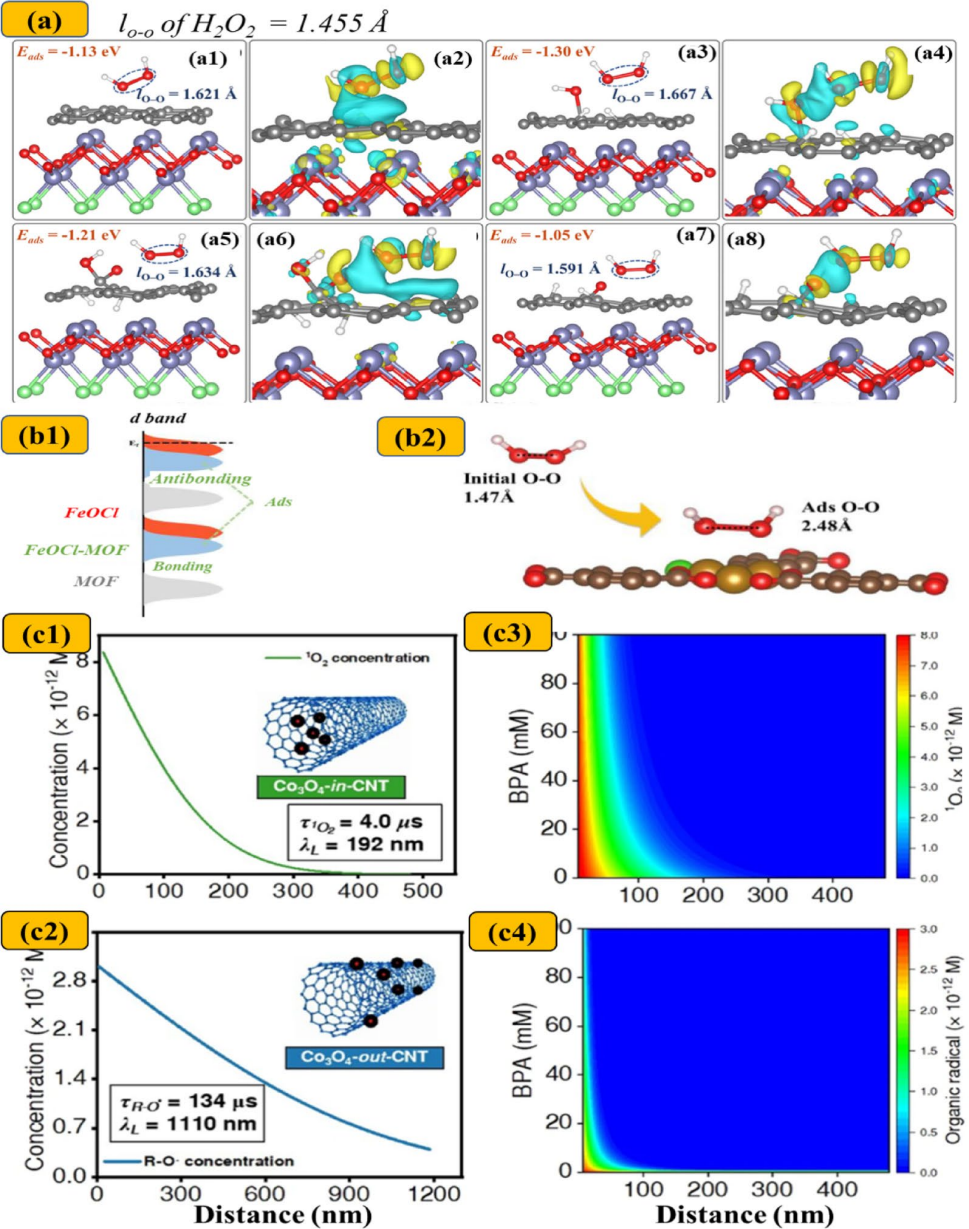
Adsorption configurations of H_2_O_2_ on the graphitic carbon surfaces functionalized with different oxygen-containing groups, **a1**–**2** FeOCl–C, **a3**–**4** FeOCl–COH, **a5**–**6** FeOCl–COOH, and **a7**–**8** FeOCl–CO, along with the corresponding charge density difference maps (yellow and cyan denote regions of electron accumulation and depletion, respectively). Reprinted with permission from [25]. Copyright (2022) American Chemical Society. **b1** Schematic illustration of the d-band center and catalyst-adsorbate bonding for FeOCl, MOF, and FeOCl@MOF, and **b2** H_2_O_2_ adsorption behavior on MIL-101. Adapted with permission from [35]. **c1**–**2** Correlation between ROS concentration and migration-diffusion distance and two-dimensional mapping of ROS exposure as a function of BPA concentration and proximity to the catalyst surface in the (**c3**) Co_3_O_4_@CNT/PAA and **c4** Co_3_O_4_/CNT/PAA systems. Reproduced from [28], licensed under CC BY 4.0

Furthermore, the abundance of adsorption sites provided by carbon-confined catalysts is believed to enhance the thermodynamic adsorption of reactants and accelerate mass transfer kinetics in chainmail catalysis. Density functional theory (DFT) has emerged as a powerful tool for quantitatively assessing the thermodynamic behavior of oxidant adsorption (Fig. 3). DFT calculations have consistently shown that carbon encapsulation lowers the adsorption energy (E_ad_) of oxidants such as PMS, PS, and H_2_O_2_, while simultaneously increasing the bond length of the peroxide bond (d_O–O_). These changes indicate more favorable adsorption thermodynamics and improved susceptibility of the oxidants to bond cleavage, facilitating the generation of ROS. For example, Yu et al. [96] observed a substantial elongation of the O–O bond in PMS upon adsorption onto Co encapsulated within nitrogen-doped graphene (Co@NG), with d_O–O_ increasing to 1.45 Å, compared to 1.31 Å for pristine PMS and 1.44 Å for PMS on pure Co, suggesting superior PMS activation in the encapsulated system [96]. In another study, H_2_O_2_ adsorption on a FeOCl/CC@rGO cathode exhibited the longest d_O–O_ bond length (1.741 Å) and the lowest E_ad_ (− 1.66 eV), in contrast to FeOCl/CC (1.621 Å, − 1.13 eV) and free H_2_O_2_ (1.455 Å), corresponding to enhanced degradation of OTC [25]. Similarly, Su et al. [88] attributed the improved performance of Fe_3_O_4_@CNTs to stronger PS binding, with an E_ad_ of − 1.70 eV compared to − 0.78 eV for Fe_3_O_4_/CNTs. The O–O bond in PS was also more elongated in the confined system (1.99 Å) than in Fe_3_O_4_/CNTs (1.84 Å) or pristine PS (1.48 Å), indicating stronger oxidant–catalyst interactions and greater efficacy in encapsulated systems [88].

Generally, an increase in active surface area is known to enhance mass transfer kinetics, thereby facilitating improved catalytic performance [17, 98]. Upon adsorption, reactants are activated to generate ROS, which may either remain adsorbed on the catalyst surface or diffuse into the bulk solution [99]. However, given their extremely short lifetimes and limited diffusion lengths, most ROS tend to react within proximity to the catalyst surface [94, 100]. Notably, prior studies have suggested that the incorporation of carbon layers on cobalt catalysts (e.g., Co@C) can enhance the exposure of surface active sites, thereby promoting ROS desorption and facilitating their diffusion into the bulk solution [50]. In a notable example, Liu et al. reported diffusion distance and lifetime of ^1^O_2_ (192 nm and 4 μs) was much lower than that of R–O• radicals (1110 nm and 134 μs) when using Co_3_O_4_@CNTs and Co_3_O_4_/CNT, respectively, during PAA activation (Fig. 3c). Despite a much lower extent, the nanoconfined structure allows extraordinary exposure of BPA with ^1^O_2_, showing 99% consumption of ^1^O_2_ within 350 nm from the surface, [28]. Nevertheless, such mechanistic insights remain limited in the current literature and have not been validated in other studies, indicating the need for further studies to better understand mass transfer behavior in chainmail catalysis systems.

It is also important to note that enhanced adsorption capacity does not always correlate with superior catalytic activity. Several studies have reported contradictory findings in this regard [30, 101, 102], emphasizing the critical role of subsequent ROS generation. It is this step, rather than adsorption alone, that ultimately drives the dominant radical and non-radical degradation pathways responsible for effective contaminant removal.

### Degradation mechanisms in chainmail catalysis

#### Radicals

Several studies have indicated that radical-based pathways dominate the degradation mechanisms of micropollutants in chainmail catalysis. For example, a recent study reported significantly elevated steady-state concentrations of •OH and SO_4_•‾ radicals, measured at 5.13 × 10^–11^ mM and 6.30 × 10^–11^ mM, respectively, when using the Co@NCNT/PMS system. These values were approximately five times higher than those observed in the unconfined Co/g-C_3_N_4_/PMS system, which exhibited corresponding concentrations of 1.09 × 10^–11^ mM and 1.15 × 10^–11^ mM, respectively [33]. Similarly, in another study, both Co_3_O_4_ and nitrogen-doped carbon (NC) alone showed negligible H_2_O_2_ activation, generating •OH at rates below 0.1 μM/min and 0.25 μM/min, respectively. In contrast, the confined Co_3_O_4_@NC catalyst significantly enhanced •OH production, achieving a rate of 2 μM/min [46]. Additionally, electron paramagnetic resonance (EPR) spectroscopy detected a significantly stronger 5,5-dimethyl-1-pyrroline-N-oxide (DMPO)–OH signal in FeCo@C cathodes compared to those containing unconfined bimetallic FeCo alloys, further confirming the superior radical generation capabilities of chainmail catalysts [44].

In contrast to studies emphasizing radical-driven degradation, several studies have reported reduced or negligible radical formation in carbon-encapsulated catalyst systems [28, 56, 61, 97, 102, 103]. For example, Su et al. [88] observed a decrease in the relative contribution of •OH from 54.7% to 39.1% when Fe_3_O_4_ was loaded on the exterior versus the interior of CNTs, respectively [88]. Similarly, Yang et al. 12 reported the absence of a detectable DMPO–OH signal (an indicator of •OH presence) in the Fe_2_O_3_@CNT/H_2_O_2_ system, suggesting minimal radical formation [56]. A negligible role of •OH and R-O• in BPA degradation was also noted in the Co_3_O_4_@CNTs/PAA system [28]. Collectively, these results indicate that radical-based degradation may not be the sole mechanism driving contaminant removal in chainmail catalysis.

#### Non-radicals

**Fig. 4:**
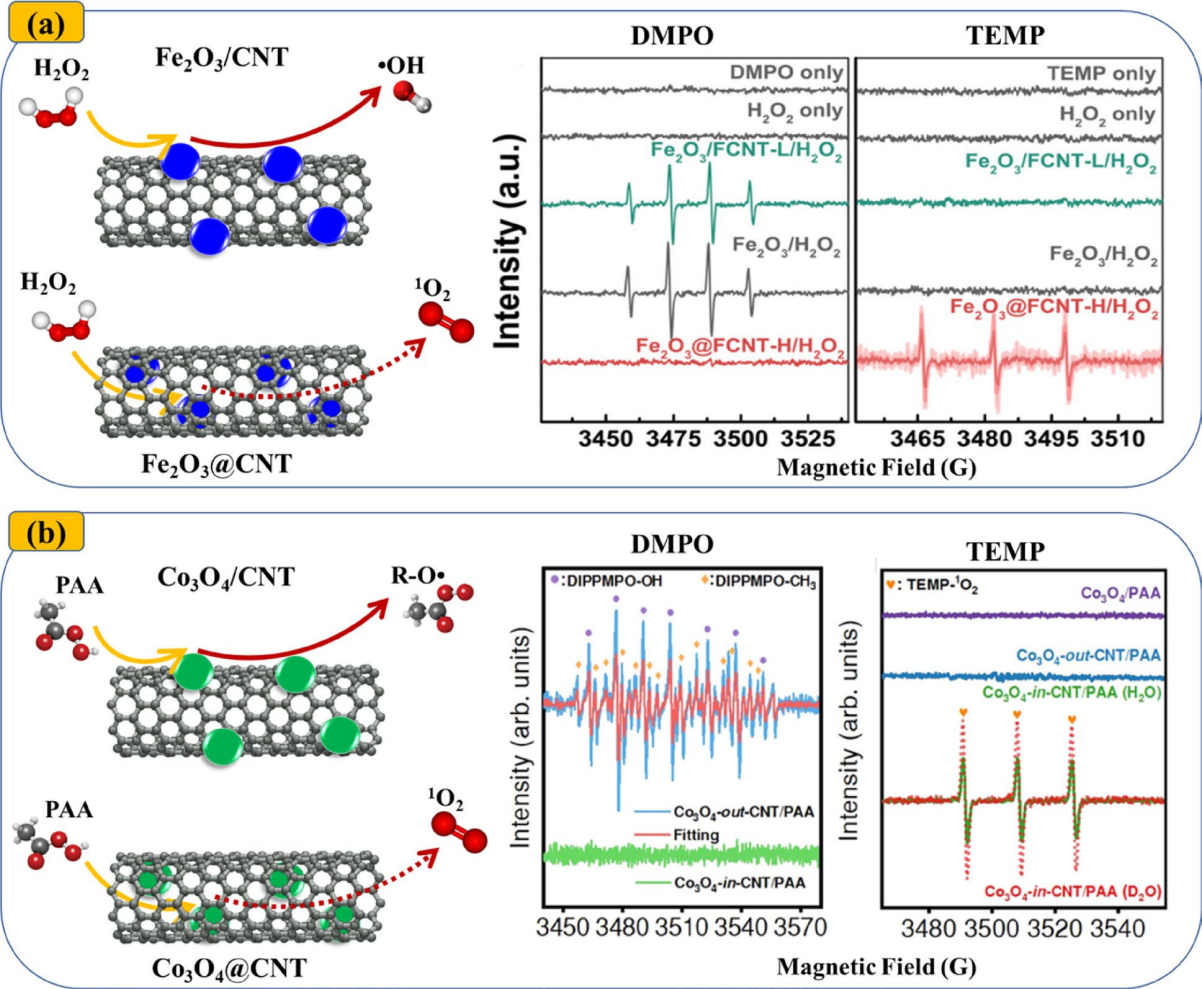
EPR spectra showing the presence of radical and non-radical species in (**a**) Fe_2_O_3_/CNT versus Fe_2_O_3_@CNT, and **b** Co_3_O_4_/CNT versus Co_3_O_4_@CNT. Reproduced from [56], licensed under CC BY-NC-ND 4.0, and [28], licensed under CC BY 4.0, respectively

Growing evidence underscores the significant role of non-radical degradation pathways in chainmail catalysis (Fig. 4). Unlike conventional AOPs, chainmail catalysts feature a carbon shell that establishes a nanoconfined interfacial environment, enabling highly regulated electrochemical reactions and promoting unconventional oxidation behavior [28, 35, 48, 50, 86].

Among non-radical reactive species, the pivotal role of ^1^O_2_ has been firmly established through EPR spectroscopy and quenching experiments. Strong EPR signals indicative of ^1^O_2_ have been consistently detected across multiple systems, including Fe_2_O_3_@CNT/H_2_O_2_ [56], Co_3_O_4_@CNT/PAA [28], Co@CNT/PMS [33], Fe_3_C/Fe@NC/PMS [97], FeCo@OCNT in EF systems [31], and Co@NC/PMS [102]. In some cases, the contribution of ^1^O_2_ to pollutant degradation has even surpassed that of radical pathways. For example, in the Fe_3_C@NCT/PS system, ^1^O_2_ was the dominant species responsible for SMX degradation, as both •OH and SO_4_•‾ rapidly diminished within the first 10 min, whereas a strong ^1^O_2_ signal persisted for up to 30 min [38]. Similarly, in the Co_3_O_4_@CNT/PMS system, the addition of 100 mM ethanol, a radical scavenger, led to only a 15% reduction in NX removal, in contrast to a 65% decrease observed upon the introduction of a ^1^O_2_ scavenger [61]. The incorporation of a carbon outer layer in chainmail catalysts underscores the critical role of electron-transfer mediation (ETM) in facilitating catalysis processes.

**Fig. 5:**
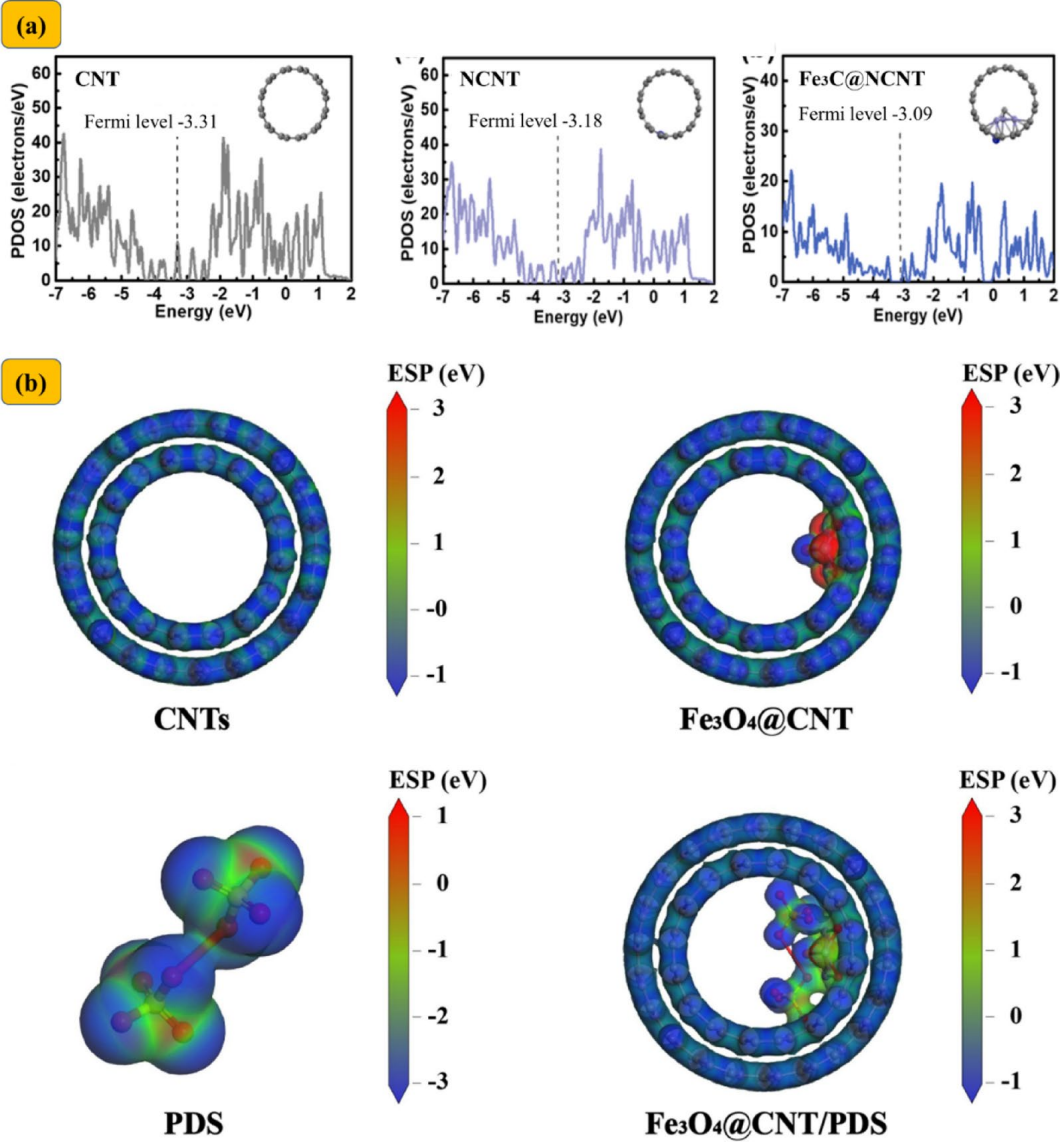
**a** Projected density of states (PDOS) for CNT, NCNT, and Fe_3_C@NCNT. Reproduced with permission from [105]. **b** Distribution and electrostatic potential for different CNT fragments via DFT calculations for CNTs, Fe_3_O_4_@CNT, PDS, and Fe_3_O_4_@CNT with adsorbed PDS. Reproduced with permission from [48]

**Fig. 6:**
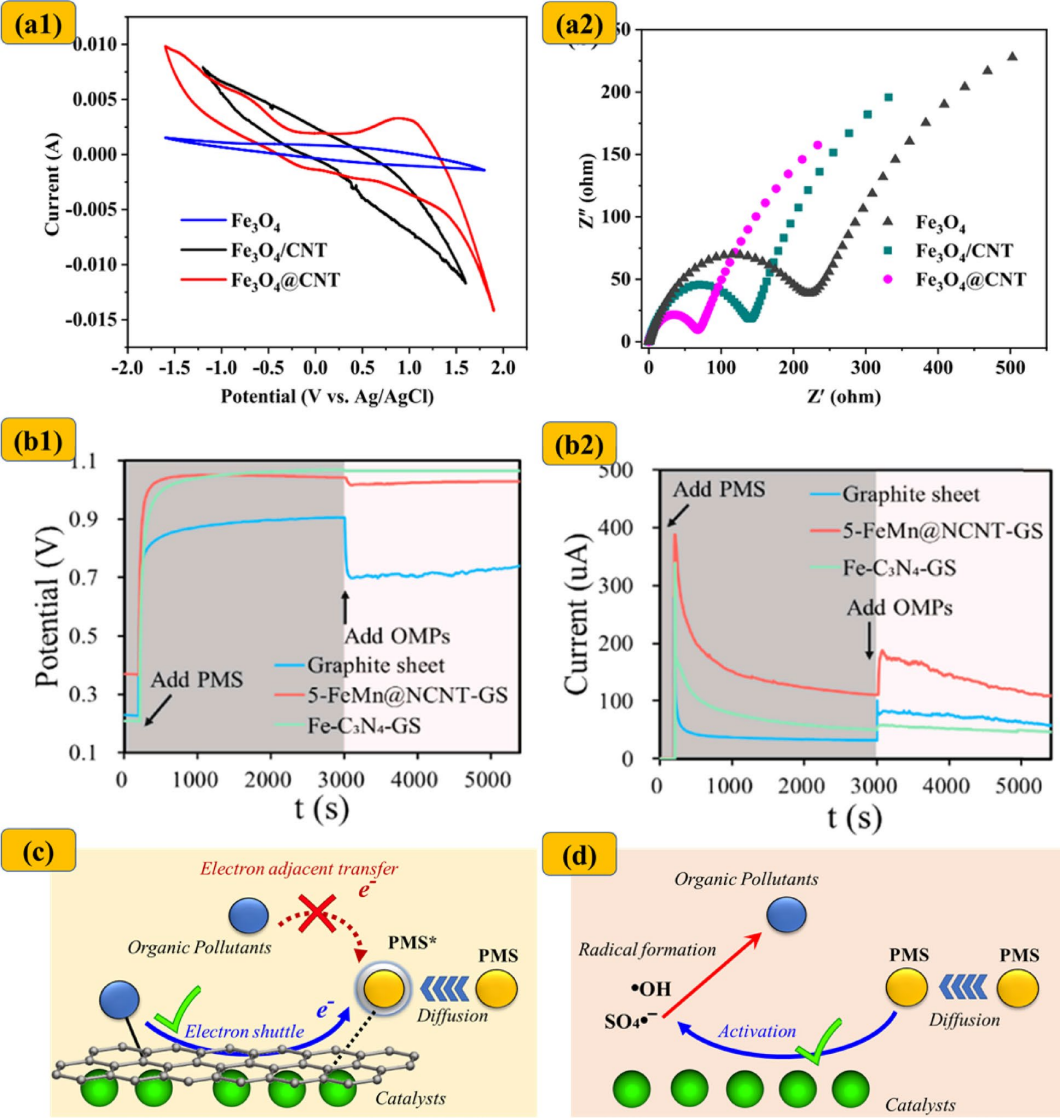
**a1** Cyclic voltammetry curves and **a2** EIS Nyquist plots comparing Fe_3_O_4_, Fe_3_O_4_/CNT, and Fe_3_O_4_@CNT. Reprinted with permission from [48]. **b1** Chronopotentiometry response and **b2** Chronoamperometry response of working electrodes with graphite only, Fe-C_3_N_4_-graphite, and 5-FeMn@NCNT following sequential addition of PMS and 4-CP, and representation of various electron transfer regimes in (**c**) chainmail catalytic systems and **d** conventional AOPs. Adapted from [30], with permission from Elsevier

Yun et al. [104] were among the first to challenge the prevailing emphasis on ^1^O_2_ in CNT/PMS systems, proposing instead that ETM may serve as the dominant degradation pathway [104]. In chainmail catalysis, ETM-driven degradation is inherently complex due to the multifaceted electrochemical interactions among transition metals, carbon layers, oxidants, and target pollutants. Nanoconfined TM/TMO catalysts are capable of transferring electrons to adjacent carbon shells through interfacial metal–carbon interactions, thereby modulating the Fermi level and enhancing the localized charge density within the carbon layer (Fig. 5) [48, 50, 105]. For example, Fe_3_C@CNT exhibited an elevated D-band center (from − 3.18 eV in bare CNTs to − 3.09 eV), reflecting improved electronic states near the Fermi level [105]. Similarly, the Fermi level of Co@NC shifted from − 0.16 eV (NC) to − 0.19 eV [102]. Comparable downshifts were observed in Co_3_O_4_@CNT (− 0.201 eV) and Fe_3_O_4_@CNT (− 0.214 eV), relative to pristine CNTs (− 1.90 eV), indicating enhanced electronic conductivity and reactivity in these carbon-confined systems [48, 61]. Electrochemical measurements further validated the superior electron-transfer properties of encapsulated TM/TMO catalysts (Fig. 6), with multiple studies reporting smaller semicircle diameters in Nyquist plots, indicating reduced charge transfer resistance compared to their bare metal counterparts [35, 47, 48, 106].

Due to their enhanced electrical conductivity, the carbon outer layers in TM/TMO–carbon hybrid catalysts function as rapid electron transport channels, thereby facilitating ETM-driven degradation of micropollutants. This mechanism is typically evidenced by a two-stage open-circuit potential (OCP) response: an initial increase in OCP upon oxidant addition, followed by a decrease after pollutant injection (Fig. 6) [30, 34, 39, 48, 61, 65, 97, 107]. For example, in the FeCo@NC/PMS system, the initial OCP rise corresponds to PMS adsorption and the formation of a high-energy PMS* intermediate. This reactive species subsequently extracts electrons from Carbamazepine (CBZ), leading to its degradation via an electron-mediated, non-radical pathway, as reflected by the subsequent decrease in OCP [65]. Duan et al. [30] provided further support for this ETM mechanism by demonstrating that electron transfer occurred primarily from the micropollutant (acting as the electron donor), through the hybrid catalyst (serving as the electron mediator), to the PMS (electron acceptor), rather than via direct pollutant-to-PMS electron transfer [30]. The crucial role of nanoconfined TM/TMO structures was further substantiated by a more pronounced OCP shift observed in Fe@NCNT compared to bare NCNT [107].

An additional non-radical degradation pathway in chainmail catalysis may involve high-valent metal–oxo species, particularly when metal cations such as Fe, Co, or Ag exist in reduced oxidation states [18]. Under specific conditions, metal–oxo-mediated oxidation can outperform conventional radical pathways due to its high redox potential, enhanced selectivity, and extended lifetime [108]. For example, iron–oxo species formed in PMS/Fe(III) systems with organic ligands [109] or supported g-C_3_N_4_ [110] have demonstrated effective degradation of 4-chlorophenol (4-CP), exhibiting strong resistance to interference by common anions (e.g., Cl‾, SO_4_‾, and NO_3_‾) and dissolved organic matter (DOM). Despite such findings, the role of high-valent metal–oxo species in chainmail catalysis remains ambiguous and often underappreciated, with studies reporting conflicting results. Some investigations, such as those involving Fe_3_C/Fe@NC/PMS [97] and FeOCl/MOF systems [35], have suggested minimal involvement of iron–oxo species. Conversely, other reports have highlighted their significant contribution to overall degradation performance [28, 53, 107]. The inconsistent observations regarding the role of metal-oxo species in overall degradation performance likely stem from several mechanistic and structural factors. The type of metal core can strongly influence the formation and thus the reactivity of high valent species, e.g., Fe-based catalysts [35, 97] versus Co-based [28] and bimetal FeMn-based catalysts [53], which lead to different metal-oxo species formation. In addition, experimental conditions could also contribute to the discrepancy. For instance, different reactants, such as H_2_O_2_, PMS, and PAA, were used in those studies, which resulted in different degradation routes. Finally, the configuration of chainmail catalysts could also play an important role in the contribution of metal-oxo species. The protective carbon shell structure not only controls the release of metals but also regulates the electron transfer mechanisms. As a result, direct oxidation of pollutants by metal–oxo species may be limited, but the existence of these species may function as intermediates in the generation of ROS [111, 112]. Further research is needed to clarify the formation, stability, and reactivity of high-valent metal–oxo species in chainmail catalytic systems.

## Key parameters affecting chainmail catalytic performance

### Catalyst structure and activity

Calcination plays a pivotal role in strengthening the interaction between the TM core and the carbon shell synthesized via hydrothermal or solvothermal methods [42]. As such, pyrolysis temperature emerges as a key parameter that significantly influences the catalytic efficiency of chainmail systems (Table 2). For example, Wu et al. reported a substantial increase in MO removal efficiency (from 27 to 96%) as the pyrolysis temperature of Co@NCs was raised from 500 to 900 ℃ [36]. Similarly, in the Co@NGs/PMS system, phenol degradation increased from 48.5% at 700 ℃ to complete removal at 900 ℃ but decreased to 87.5% at 1100 ℃ [96]. A comparable trend was observed for Orange II removal using Fe@C-BN/PMS: as the calcination temperature increased from 700 to 800 ℃, performance improved (k_obs_ = 0.02 to 0.058 min^−1^), but further elevation to 1000 ℃ reduced efficiency (k_obs_ = 0.004 min^−1^) [113]. These effects are primarily attributed to two structural and compositional changes induced by temperature. First, calcination affects the graphitization and structural integrity of the carbon layers. Enhanced catalytic performance at higher temperatures is often linked to improved graphitic structure, as indicated by a lower D band to G band intensity ratio (I_D_/I_G_). However, excessive thermal treatment can compromise the carbon framework, leading to a decrease in activity [38, 55, 90, 96, 103, 114]. For example, Yu et al. [96] found that nitrogen content was higher in Co@NG synthesized at 900 ℃ than at 1100 ℃, attributing this to the lower bond dissociation energy of the C–N bond (305 kJ mol^−1^) compared to the C–C bond (379 kJ mol^−1^) [96]. Second, calcination temperature directly affects the chemical composition of metal cores. Yang et al. [62] demonstrated that Fe@C calcinated at 800 °C achieved superior removal of Monochlorobenzene (MCB), attributed to the greater reduction of Fe^3+^ to Fe^0^, in contrast to the presence of Fe_2_O_3_ and FeO at 400 ℃ and 600 ℃, respectively. In a separate study, Fe_3_O_4_ remained after calcination at 500 ℃, while complete conversion to Fe^0^ was observed at 700 ℃ [63]. A positive correlation was also identified between the relative abundance of Fe/Fe_3_C phases and increasing pyrolysis temperature [115], suggesting that TM composition in carbon-encapsulated catalysts can be deliberately tuned through thermal treatment. Therefore, identifying the optimal calcination temperature is essential for maximizing the catalytic performance of carbon-encapsulated TM catalysts.

**Table 2:** Impact of pyrolysis temperature on the removal performance of catalysts

Pyrolysis temp	Catalysts	Reactants	Micropollutants	Observations	Reference
500–900 ℃	FeMn@NC 0.2 g/L	PMS 0.5 mM	SMZ 10 mg/L	RR↑ from 500 (0.023 min^−1^) to 800 (0.111), then ↓ at 950 (0.03 min^−1^)	[53]
700–900 ℃	Fe_3_C/Fe@NC 0.3 g/L	PMS 0.1 mM	SMX 20 mg/L	RR↑ from 700 (1.8 min^−1^) to 900 (2.84 min^−1^)	[97]
700–900 ℃	Co@MOC 0.16 g/L	PMS 1.3 mM	TC 20 mg/L	RR↑ from 700 (0.58) to 800 (0.871), then ↓ at 900 (0.756 min^−1^)	[116]
600–900 ℃	Co@NCNT 0.05 g/L	PMS 4 g/L	TC 30 mg/L	RR↑ from 600 (0.04 min^−1^) < 700 (0.049) < 800 (0.066) then ↓ at 900 (0.046 min^−1^)	[114]
700–1000 ℃	Co–N@NC 0.1 g/L	PMS 0.1 g/L	SMX 20 mg/L	RR↑ from 700 (68.38%) to 1000 (99.25%)	[54]
700–900 ℃	Fe/Fe_3_C@NCNT 0.6 g/L	H_2_O_2_ 9 mM	NOR 0.063 mM	RR↑ from 700 (0.015 min^−1^) to 800 (0.027), then ↓ at 900 (0.021 min^−1^)	[105]
700–1000 ℃	FeMn@NCNT 0.1 g/L	PMS 1 g/L	4-CP 0.05 mM	RR↑ from 700 (0.334 min^−1^) to 900 (0.692), then ↓ at 1000 (0.147 min^−1^)	[30]
700–1100 ℃	Co@NG 0.05 g/L	PMS 3 mM	Phenol 1 mM	RR↑ from 700 (0.045 min^−1^) to 900 (0.397), then ↓ at 1000 (0.218), 1100 (0.161 min^−1^)	[96]
600–900 ℃	Co@PCN 0.1 g/L	PMS 0.6 mM	CIP 15 mg/L	RR↑ from 600 (41.3%) to 900 (90.1%)	[117]
400–800 ℃	Mn_3_O_4_@C 0.2 g/L	PS 2 g/L	2,4-D 100 mg/L	RR↓ from 400 (0.0175 min^−1^) to 800 (0.011 min^−1^)	[90]
300–700 ℃	Fe_0_@C 0.25 g/L	PS 1 mM	4-CP 0.15 mM	RR↑ from 300 (10%) to 700 (82%)	[63]
600–1000 ℃	Fe_3_C@NCNT 0.1 g/L	PS 1 mM	SMX 5 mg/L	RR↑ from 600 (0.01 min^−1^) to 900 (0.089), then ↓ at 1000 (0.065 min^−1^)	[38]
600–1050 ℃	FeMn@NCNT 0.1 g/L	PMS 6.5 mM	Acetamiprid 7.8 μM	RR↑ from 600 (0.006 min^−1^) to 900 (0.064), then ↓ at 1050 (0.053 min^−1^)	[39]
500–700 ℃	Co@NCNT 0.4 g/L	SO32- 5 mM	MO 20 mg/L	RR↑ from 500 (27%) to 700 (96%)	[36]
700–900 ℃	Fe_3_C@NCNT 0.2 g/L	PMS 0.2 g/L	Phenol 20 mg/L	RR↑ from 700 (0.097 min^−1^) to 900 (0.330 min^−1^)	[103]
700–1000 ℃	Fe@C-BN 20 mg/L	PMS 0.1 g/L	Orange II 20 mg/L	RR↑ from 700 (0.02 min^−1^) to 800 (0.058), then ↓ at 900 (0.011), 1000 (0.004 min^−1^)	[113]

### Carbon shell morphology and thickness

**Fig. 7:**
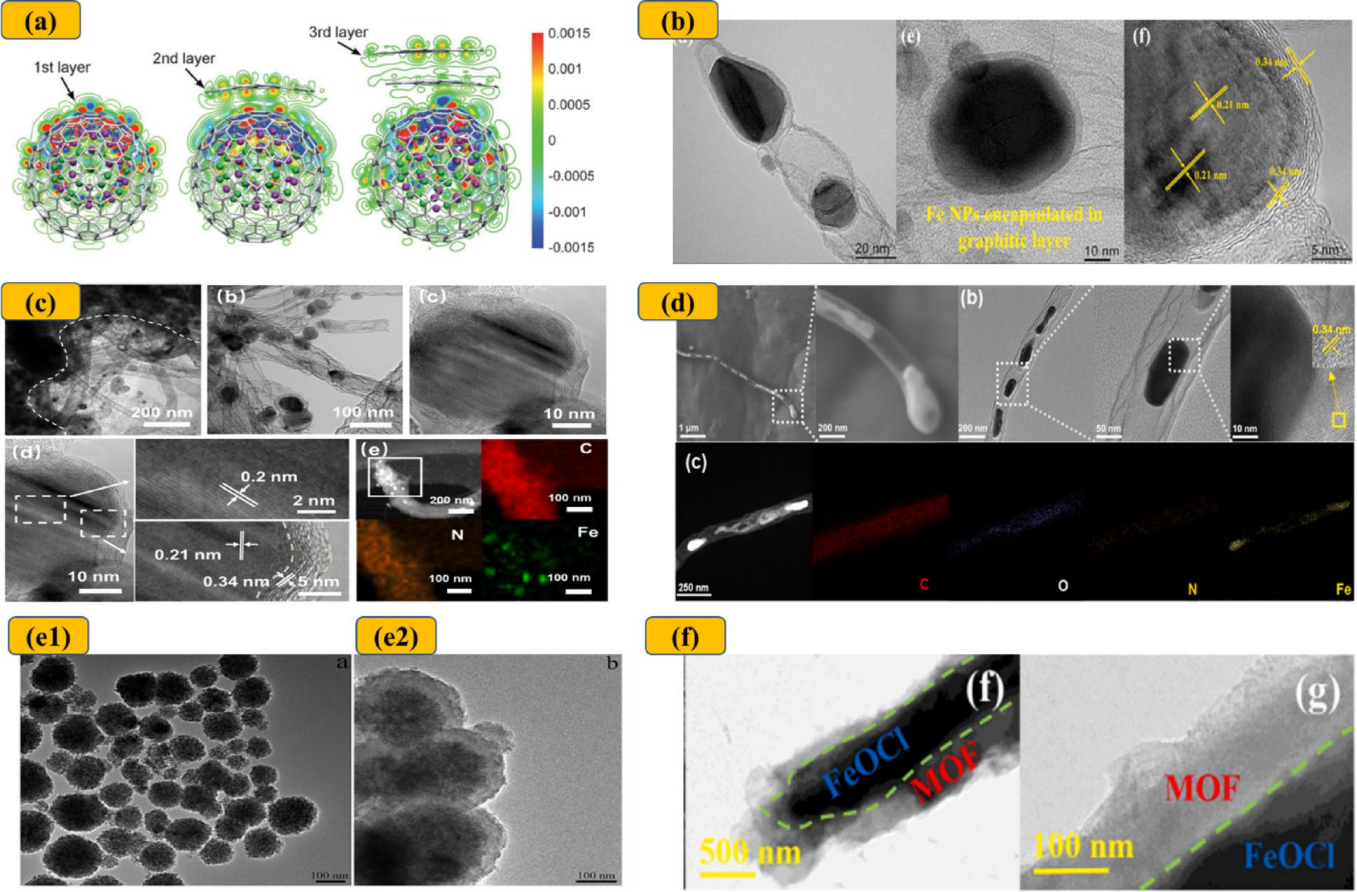
**a** Electron density redistribution upon encapsulation of a CoNi cluster with one to three layers of graphene. Red and blue regions represent increased and decreased electron density, respectively. Reproduced with permission [118]. Copyright 2015, Wiley–VCH. HRTEM and SEM images of catalysts encapsulated within ultrathin graphene layers: **b** Fe_3_C/Fe@NC-9. Reproduced with permission from [97], **c** Fe/Fe_3_C@NCNT Reproduced with permission from [105], and **d** Fe@NCNT. Reproduced with permission from [107]. **e** TEM images of catalysts confined within thick carbon layers: **e1** CuFe_2_O_4_, **e2** CuFe_2_O_4_@PDA. Reproduced with permission from [40], and **f** FeOCl@MOF. Reproduced with permission from [35]

The thickness of the external carbon shell is widely regarded as one of the most critical parameters in chainmail catalysis. Deng et al. [26] were the first to propose that the catalytic effectiveness of chainmail structures depends on maintaining carbon layer thicknesses of fewer than three layers (approximately 5 nm), to enable efficient electron transfer across the carbon framework (Fig. 7) [26]. This conclusion has since been broadly acknowledged and corroborated by several studies [25, 36, 38, 39, 47]. However, emerging evidence suggests that chainmail catalysis can remain effective even with substantially thicker carbon layers [28, 32, 35, 40]. For example, Zuo et al. [35] demonstrated enhanced BPA removal using FeOCl confined within a 100 nm MOF layer compared to bare FeOCl, despite reduced electron transfer efficiency. This improvement was attributed to the facilitated desorption of •OH radicals within the FeOCl–MOF structure, which mitigates in situ radical quenching and prevents active site poisoning in hybrid materials [35]. These findings suggest that while a thinner carbon shell can enhance electron-mediated processes, effective catalysis may still occur at greater thicknesses through alternative mechanisms.

In the context of nanocatalyst confinement within one-dimensional (1D) carbon nanotubes (CNTs), the diameter of the carbon shell emerges as a key factor influencing catalytic performance. The inherent curvature of CNTs, which is defined by their diameter, leads to a redistribution of π-electron density from the inner concave surface to the outer convex surface, thereby establishing an electric potential gradient [64]. Unlike two-dimensional (2D) sp^2^-hybridized graphene, curved 1D CNTs exhibit enhanced chemical reactivity due to the partial incorporation of s-electron character into their π orbitals. This reactivity is further amplified by structural defects, such as vacancies, non-six-membered rings, zigzag edges with unpaired π electrons, and other functional groups present at defect sites in CNTs [119]. As the diameter of CNTs decreases, spatial confinement increases, resulting in smaller, sub-nanometer-sized metal particles and stronger interfacial interactions between the encapsulated metal catalyst and the graphitic shell [120]. These observations were corroborated in a subsequent study involving Fe@CNT systems [121]. However, stronger metal–carbon interactions do not always equate to improved catalytic degradation. For example, nearly complete phenol degradation was achieved using CNTs with a 10 nm diameter, surpassing the 90.6% removal efficiency observed with narrower 3 nm CNTs. This counterintuitive trend may stem from several factors, including lower redox potentials of the synthesized core catalysts, spatial limitations that hinder reactant diffusion, and restricted formation of ROS in smaller-diameter cavities [121].

In addition to 1D CNTs, MOF-derived core–shell materials have been widely investigated for their potential in chainmail catalysis. These materials are typically synthesized through coordination between metal salt precursors and organic ligands, followed by thermal calcination [31, 34, 35, 53, 54, 96, 102, 122, 123]. For example, Liu et al. employed a combination of a zinc imidazolate framework (ZIF-8), cobalt nitrate, and dopamine to synthesize Co–N@NC for PMS activation, achieving over 96% removal of SMX (20 mg/L) within 90 min [54]. The high catalytic efficiency was largely attributed to the three-dimensional (3D) architecture of the material, which offers a high surface area, tunable porosity, and uniformly dispersed metal active sites, features that collectively provide an optimal platform for nanoconfined catalyst stabilization and activity enhancement [42, 66]. Looking ahead, advancing the design of MOF-derived chainmail catalysts will require systematic optimization of synthesis parameters, including the choice of organic linkers, stabilizers, surfactants, solvents, and metal salt precursors, to fine-tune structural and electronic properties for maximized catalytic performance.

### Heteroatom doping and surface functionalization

Heteroatom doping involves the substitution of C atoms within the carbon lattice with other elements, offering a dual advantage: the creation of additional active sites for reactant activation and the modulation of the electronic properties of the material [124, 125]. Among various dopants, the N atom is particularly favored due to its comparable atomic radius and higher electronegativity (χ = 3.04 compared to 2.55 for the C atom), which enables its facile incorporation into the carbon framework and alters the local electronic environment of adjacent carbon atoms [119, 124]. As a result, N-doped configurations (especially TM–N–C structures) have garnered significant attention for their ability to promote efficient charge transfer from the encapsulated metal core to the carbon surface, thereby significantly improving catalytic degradation performance [43, 126].

Carbon-encapsulated catalysts enriched with graphitic and pyridinic N are generally favored, as these bonding configurations are known to lower adsorption barriers and facilitate electron transfer, thereby enhancing micropollutant degradation [38, 52–54, 67, 97, 119]. Numerous studies have reported a strong correlation between high graphitic N content and superior catalytic performance, as observed in systems such as Fe@C–PDA/PS [52], Co@NC/PMS [117], and FeMn@NC/PMS [53]. This is further supported by post-reaction analyses showing diminished peak intensities for graphitic and pyridinic N, suggesting their active involvement during catalysis [37, 39, 54, 113]. DFT calculations also revealed that PS exhibits a stronger binding affinity to graphitic N (adsorption energy =  − 0.437 eV) compared to pyrrolic N (adsorption energy =  − 0.304 eV), reinforcing the superior reactivity of graphitic N sites [38]. In contrast, the catalytic effects of other heteroatoms, such as P, S, and B, remain largely unexplored. For example, Yao et al. [37] synthesized a boron- and nitrogen-co-doped Fe@C catalyst (Fe@C–BN) that demonstrated efficient PMS activation, achieving 95% removal of Orange II within 60 min [113]. However, the specific mechanistic role of boron in the system was not thoroughly elucidated. This highlights a significant knowledge gap regarding the role of various heteroatom dopants in chainmail catalysis, warranting further investigation.

In parallel with heteroatom doping, surface functional groups also act as active sites for reactant activation. For example, functionalizing MnFe_2_O_4_@C with –NH_2_ groups tripled the catalytic degradation rate of OFX, increasing from 0.0078 to 0.0243 min^− 1^. This enhancement was attributed to increased electron density in the carbon shell, which promoted Fe^2+^ formation and facilitated Fenton-like reactions [51]. However, the catalytic influence of surface functional groups appears to be system-dependent, depending on the type of functional group and the overall catalyst configuration. For example, the O–O bond length in PS was longest in Mn_3_O_4_@C–OH (1.524 Å), followed by Mn_3_O_4_@C=O (1.449 Å) and Mn_3_O_4_@C (1.419 Å), suggesting that hydroxyl groups (C–OH) were more effective than carbonyls (C=O) in facilitating PS activation [90]. Zhao et al. (2023) observed a strong positive correlation (R^2^ = 0.81) between the combined content of C=O and carboxyl (O–C=O) groups and the degradation rate of SMX in Fe_3_C/C@NC/PMS systems [97]. Similarly, in Fe@NC/PMS systems, the CBZ degradation rate positively correlated with the sum of O–C=O and M=O group content (R^2^ = 0.942) and negatively with the content of C–O groups (R^2^ = 0.966) [65]. Expanding on this, Li et al. [114] demonstrated that different oxygen-containing functional groups selectively modulate ROS generation in Co@NC/PMS systems: C=O groups promote both ^1^O_2_ and •OH formation; C–O groups enhance •OH but suppress SO_4_•‾; and O–C=O groups significantly facilitate ^1^O_2_ production [117].

### Metal core composition and redox state

Given the critical role of interfacial interactions between the carbon shell and the inner metal core in chainmail catalysis, the composition of the TM core emerges as a key determinant of micropollutant degradation efficiency (Table 3) [84]. Under comparable catalyst loadings, degradation efficiencies for organic micropollutants were reported to be 10%, 13%, and 27% for Mn@NC, Fe@NC, and Cu@NC, respectively, substantially lower than the 96% achieved by Co@NC [36]. In a related study, Yao et al. [113] observed superior removal performance using Co@NC compared to Fe@NC and Ni@NC, despite no notable structural differences among the catalysts. This led the authors to attribute the enhanced activity to the synergistic interaction between specific TM species and N–C moieties, which facilitated more effective PMS activation [37]. In single-metal systems, it is generally accepted that TMs in lower oxidation states tend to exhibit superior catalytic activity. For example, Su et al. [89] reported a significantly higher degradation rate of phenol using Fe^0^@CNT (k_obs_ = 0.199 min^− 1^) compared to iron oxides confined within CNTs (9.3 × 10^− 4^ min^− 1^) [89]. These findings are consistent with those of another study, which demonstrated complete phenol degradation within 10 min using Co@C, whereas Co_3_O_4_@C achieved only 11.5% removal under the same conditions [50]. Collectively, these results strongly support the conclusion that reduced-state TMs offer superior catalytic performance in chainmail systems.

**Table 3:** Impact of inner core catalysts on removal performance

Materials	Reactants	Micropollutants	Observations (unit for reaction rate: min^−1^)	Reference
Co@NCNTs, Fe@NCNT, Ni@NCNT 5 mg/L	PMS	AO7 15 mg/L	RR follows order of Co@NCNT (0.712) > Fe@NCNT (0.02) > Ni@NCNT (0.006)	[84]
FeMn@NC, Fe@NC, Mn@NC, FeMn/C 0.2 g/L	PMS 0.5 mM	SMZ 10 mg/L	RR follows order of FeMn@NC (0.111) > FeMn/C (0.049) > Fe@NC (0.053) > Mn@NC (0.017)	[53]
FeOCl, MOF MIL-101 (Fe), FeOCl-MOF 0.5 g/L	H_2_O_2_ 0.5 mM	BPA 0.05 mM	RR follows order of FeOCl-MOF (0.1125) > FeOCl (0.032) > MOF (0.01)	[35]
FeCo@NC, Fe@NC, Co@NC	PMS 1.5 mM	CBZ 10 mg/L	RR follows order of FeCo@NC (0.081) > Co@NC (0.021) > Fe@NC (0.014)	[65]
Fe/Fe_3_C@NCNTs, Co@NCNT, Mo_2_C@NCNT 0.6 g/L	H_2_O_2_ 9 mM	NOR 0.063 mM	RR follows order of Fe/Fe_3_C@NCNT (93.8%) > Co@NCNT (92.1%) > Mo_2_C@NCNT (88.4%)	[105]
FeMn@NCNT, Fe@NCNT, Mn@NCNT 0.1 g/L	PMS 1 g/L	4-CP 0.05 mM	RR follows order of FeMn@NCNT (0.692) > Fe@NCNT (0.453) > Mn@NCNT (0.353)	[30]
FeCo@C, Fe@C, Co@C	EF	CIP	RR follows order of FeCo@C (1.44) > Fe^2+^ (0.11 min-1) > Fe@C (~ 0.065) > Co@C (0.04)	[44]
Fe^0^@CNT, Fe_3_O_4_/Fe^0^/CNT, Fe_3_O_4_@CNT, Fe_3_O_4_/CNT 0.2 g/L	PS 1 mM	SMT 10 mg/L	RR follows order of Fe^0^@CNT (0.061) > Fe_3_O_4_/CNT (0.023) > Fe_3_O_4_/Fe^0^/CNT (0.018) > Fe_3_O_4_/CNT (0.017)	[88]
MnFe_2_O_4_@C-NH2, MnFe_2_O_3_@C, MnFe_2_O_3_ 1 g/L	H_2_O_2_ 29.4 mM	OFX 30 mg/L	RR follows order of MnFe_2_O4@C-NH_2_ (0.0243) > MnFe_2_O_4_@C (0.0078) > MnFe_2_O_4_ (0.0048)	[51]
Fe_2_O_3_@CNT, Fe_2_O_3_/CNT 0.015 g/L, Fe_2_O_3_ 0.1 g/L	H_2_O_2_ 50 mM	MB 0.01 mM	RR follows order of Fe_2_O_3_@CNT (0.052) > Fe_2_O_3_/CNT (0.002) > > Fe_2_O_3_	[56]
Co@C, CoFe@C, CoFeP@C, CoFeO@C, Co_3_O_4_@C 0.5 g/L	PMS 0.5 g/L	Phenol 20 mg/L	RR follows order of Co@C (100%) > CoP@C (98.1%) > Co_3_O_4_@C (11.5%)	[50]
FeMn@NCNT, Fe@NCNT 0.1 g/L	PMS 6.5 mM	Acetamiprid 7.8 μM	RR follows order of FeMn@NCNT (0.064) > Fe@NCNT (0.055)	[39]
Fe@NCNT, Ni@NCNT, Co@NCNT 20 mg/L	PMS 0.65 mM	OII 20 mg/L	RR follows order of Co@NCNT (100%) > Fe@NCNT (100%) > Ni@NCNT (47%)	[37]

Across all Fenton-like mechanisms, the regeneration of metals from higher to lower oxidation states is widely recognized as a rate-limiting step, posing a significant challenge to the long-term application of these systems. One promising approach to address this limitation involves the design of encapsulated catalysts incorporating bimetallic alloys [42, 127]. Chainmail catalysts featuring dual TM cores consistently demonstrate superior micropollutant degradation performance compared to their single-metal counterparts [30, 39, 53, 55, 65, 122]. For example, in the FeMn@NCNT system, the presence of Mn facilitated the reduction of Fe^3+^ to Fe^2+^, resulting in a significantly enhanced 4-CP degradation rate relative to Fe@NCNT and Mn@NCNT alone [30]. This enhanced reactivity in bimetallic alloys is often attributed to synergistic electronic effects and an increased abundance of catalytically active sites. A recent study highlighted that FeCo@NC, characterized by a high density of M=O functional groups, significantly reduced the activation energy for O–O bond cleavage in PMS. This facilitated greater ROS generation for CBZ degradation when compared to single-core Co@NC and Fe@NC systems [65]. Importantly, the metal ratio within bimetallic systems also plays a crucial role in determining catalytic performance. In the FeCo@NC/PMS system, the k_obs_ of CBZ varied with the Fe:Co ratio, reaching 0.03, 0.09, and 0.05 min^−1^ at ratios of 2:1, 1:1, and 1:2, respectively [65]. These findings underscore the importance of precise tuning of bimetallic composition to maximize catalytic efficiency.

## Effects of operating conditions and water matrix components

### Selection of oxidants and their activation efficiency

Reactants play a pivotal role in the generation of ROS, thereby influencing both the degradation pathways and overall removal efficiency in catalytic systems (Table 4). In chainmail catalysis, oxidant activation generally follows the order of PMS > PS > H_2_O_2_, a trend observed consistently across various studies regardless of the encapsulated TMs [28, 37, 67, 90, 96, 113]. For example, PMS enabled nearly complete degradation of Orange II when activated by Fe@C-BN, significantly outperforming PS (~ 40%) and H_2_O_2_ (< 10%) under identical conditions [113]. Similarly, Mn_3_O_4_@C–T4 achieved 100% removal of 2,4-Dichlorophenol (2,4-D) within 80 min using PMS, whereas PS and H_2_O_2_ achieved only 80% and ~ 45% removal, respectively [90]. The enhanced efficacy of PMS activation is often attributed to its asymmetrical molecular structure, which facilitates more efficient bond cleavage and ROS generation [21]. In contrast, the limited effectiveness of H_2_O_2_ may be explained by its higher peroxide bond dissociation energy (E_O–O, H2O2_ = 213 kJ mol^−1^ vs. E_O–O, PS_ = 140 kJ mol^−1^), as well as its lower affinity for adsorption onto carbonaceous surfaces [96, 128, 129].

**Table 4:** Impact of operation conditions on the removal performance of chainmail catalysis

Operation conditions	Materials	Reactants	Micropollutants	Observations	Reference
*Catalyst dose*					
0–0.8 g/L	CuFe_2_O_4_@C	PS 0.1 mM	TC 50 mg/L	RR↑ from 0 to 0.6 g/L (95.9%) then ↓ at 0.8 g/L (~ 85%)	[34]
0–0.4 g/L	FeMn@NC	PMS 0.5 mM	SMZ 10 mg/L	RR↑ from 0 to 0.3 g/L (0.134 min^−1^) then ↓ at 0.4 g/L (0.105 min^−1^)	[53]
0.4–1 g/L	FeOCl@MOF	H_2_O_2_ 0.5 mM	BPA 0.05 mM	RR↑ from 0.4 g/L (0.017 min^−1^) to 1 g/L (0.055 min^−1^)	[35]
0.08–0.32 g/L	Co@MOC	PMS 1.3 mM	TC 20 mg/L	RR↑ from 0.08 (0.362) to 0.16 g/L (0.871), then ↓ at 0.24 (0.823), and 0.32 (0.136 min^−1^)	[116]
10–50 mg/L	Cu@G	PAA 0.2 mM	SMT 0.01 mM	RR increases with catalyst loading from 10 (87.1%) to 20 mg/L (92.5%), then remains stable at 30–50 mg/L	[131]
0.05–0.3 g/L	Co@NCNT	PMS 0.4 g/L	TC 30 mg/L	RR↑ from 0.05 (92%) to 0.3 g/L (99%)	[114]
0.05- 2 g/L	Co_3_O_4_@CNT	PAA 0.2 mM	BPA 0.1 mM	RR↑ from 0.05 (0.118 min^−1^) to 0.2 (0.748 min^−1^)	[28]
0.02—0.2 g/L	Co@NC	PMS 0.75 mM	TC 0.05 mM	RR↑ from 0.02 (90%) to 0.05 (95%), then decrease at 0.2 (~ 85%)	[102]
0.1–0.8 g/L	Fe_3_O_4_@CNT	PS 0.5 mM	TL 8 mg/L	RR↑ from 0.1 (61.6%) to 0.4 g/L (98.1%), then ↓ at 0.8 g/L (82.7%)	[48]
0.1–0.4 g/L	Mn_3_O_4_@C	PS 2 g/L	2,4-D 100 mg/L	RR↑ from 0.1 (0.0089 min^−1^) to 0.4 g/L (0.023 min^−1^)	[90]
0.1–0.3 g/L	Fe@C-PDA	PS 0.2 g/L	TC 100 mg/L	RR↑ from 0.1 (~ 90%) to 0.3 (~ 99%)	[52]
0- 0.35 g/L	CuFe_2_O_4_@PDA	H_2_O_2_ 0.25 M	MB 10 uM	RR↑ from 0 to 0.2 g/L (~ 90%) then stable to 0.35 g/L (~ 90%)	[40]
*Reactant dose*					
0–2 mM	CuFe_2_O_4_@C 0.4 g/L	PS	TC 50 mg/L	RR↑ from 0 mM (80%) to 1 mM (~ 95%), then ↓ at 2 mM (90%)	[34]
0.15–0.75 mM	FeMn@NC 0.2 g/L	PMS	SMZ 10 mg/L	RR↑ from 0.15 (0.008 min^−1^) to 0.75 mM (0.138 min^−1^)	[53]
0.3–1 mM	FeOCl@MOF 0.5 g/L	H_2_O_2_	BPA 0.05 mM	RR↑ from 0.3 (0.022 min^−1^) to 1 mM (0.062 min^−1^)	[35]
0.7–1.6 mM	Co@MOC 0.16 g/L	PMS	TC 20 mg/L	RR↑ from 0.7 mM (0.608 min^−1^) to 1.3 mM (0.871), then ↓ at 1.6 mM (0.648 min^−1^)	[116]
0.1–0.5 mM	Cu@G 0.02 g/L	PAA	SMT 0.01 mM	RR↑ from 0.1 mM (87.7%) to 0.5 mM (100%), then stable at 1 mM (100%)	[131]
0.1–0.5 g/L	Co@NCNT 0.05 g/L	PMS	TC 30 mg/L	RR↑ from 0.1 g/L (~ 90%) to 0.4 g/L (~ 95%) then ↓ at 0.5 g/L (93.41%)	[114]
10–1000 uM	Co_3_O_4_@CNT 0.1 g/L	PAA	BPA 0.1 mM	RR↑ from 10 uM (~ 0.02 min^−1^) to 1000 uM (~ 0.36 min^−1^)	[28]
0.05–0.2 g/L	Co–N@NC 0.1 g/L	PMS	SMX 20 mg/L	RR↑ from 0.05 g/L (70%) to 2 g/L (99%)	[54]
0.5–2 mM	FeCo@NC	PMS	CBZ 10 mg/L	RR↑ from 0.5 mM (0.019 min^−1^) to 1.5 mM (0.081), then ↓ at 2 mM (0.071 min^−1^)	[65]
6–21 mM	Fe/Fe_3_C@NCNT 0.6 g/L	H_2_O_2_	NOR 0.063 mM	RR↑ from 6 to 9 mM, then ↓ at 15 mM	[105]
0.5–2 g/L	FeMn@NCNT 0.1 g/L	PMS	4-CP 0.05 mM	RR↑ from 0.5 g/L (0.382 min^−1^) to 2 g/L (0.798 min^−1^)	[30]
0.05–0.3 g/L	Ni@NC 0.1 g/L	PS	TC 20 mg/L	RR↑ from 0.05 g/L (~ 87%) to 0.1 g/L (96.1%) then ↓ at 0.2–0.3 g/L (~ 94%)	[132]
0.1–2 mM	Fe_3_O_4_@CNT 0.2 g/L	PS	TL 8 mg/L	RR↑ from 0.1 mM (0.02 min^−1^) to 1 mM (0.099), then ↓ at 2 mM (0.052 min^−1^)	[48]
0.5–8 mM	Fe_3_C@NCNT 0.1 g/L	PS	SMX 5 mg/L	RR↑ from 0.5 mM (0.043 min^−1^) to 8 mM (0.055 min^−1^)	[38]
0.33–2.60 mM	NiCo@NCNT 0.05 g/L	PMS	IBP 20 mg/L	RR↑ from 0.33 mM to 1.3 mM, then ↓ at 2.60 mM	[55]
0.1–0.5 g/L	Fe^0^/Fe_3_C@NC 0.2 g/L	PS	BPA 50 mg/L	RR↑ from 0.1 g/L (0.003 min^−1^) to 0.5 g/L (0.01 min^−1^)	[134]
0.02–0.14 g/L	Fe@C-BN 20 mg/L	PMS	OII 20 mg/L	RR↑ from 0.02 g/L to 0.1 g/L then stable at 0.14 g/L	[113]
*Reactant type*					
PAA 0.2 mM, H_2_O_2_ 0.11 mM	Cu@G 20 mg/L		SMT 0.01 mM	RR follows order PAA (0.062) > H_2_O_2_ (0.001)	[131]
PAA, PMS, PS, H_2_O_2_ 0.2 mM	Co_3_O_4_@CNT 0.1 g/L		BPA 0.1 mM	RR follows order PAA (95.8%) > PMS (72.5%) > PS (61.4%) > H_2_O_2_ (31.4%)	[28]
PMS 3 mM, PS 3 mM, H_2_O_2_ 3 mM	Co@NG 0.05 g/L		Phenol 1 mM	RR follows order PMS (100%) > PS (34%) > H_2_O_2_ (< 5%)	[96]
PMS, PS, H_2_O_2_ 1 mM	Fe^0^@CNT 0.2 g/L		SMT 10 mg/L	RR follows order PS (99%) > PMS (87%) > H_2_O_2_ (70%) at pH 7	[88]
PMS 8.4 mM, PS 2 g/L, H_2_O_2_ 8.4 mM	Mn_3_O_4_@C 0.2 g/L		2,4-D 100 mg/L	RR follows order PMS (100%) > PS (95%) > H_2_O_2_ (72%)	[90]
PMS, PS, H_2_O_2_ 40 mg/L	Fe–N@C 40 mg/L		OII 20 mg/L	RR follows order PMS (~ 95%) > PS & H_2_O_2_ (negligible catalytic degradation)	[67]
PMS, PS, H_2_O_2_ 0.1 g/L	Fe@C-BN 20 mg/L		OII 20 mg/L	RR follows order PMS (94.5%) > PS (~ 55%) > H_2_O_2_ (< 5%)	[113]
PMS, PS, H_2_O_2_ 0.65 mM	Co@NC 20 mg/L		OII 20 mg/L	RR follows order PMS (100%) > PS (~ 65%) > H_2_O_2_ (< 10%)	[37]
*Pollutant concentration*					
0.02–0.10 mM	FeOCl@MOF 0.5 g/L	H_2_O_2_ 0.5 mM	BPA	RR↓ from 0.02 mM (0.036 min^−1^) to 0.1 mM (0.022 min^−1^)	[35]
10–40 mg/L	Co–N@NC 0.1 g/L	PMS 0.1 g/L	SMX	RR↓ from 10 mg/L (99.27%) to 40 mg/L (~ 70%)	[54]
0.025–0.15 mM	Co@NC 0.025 g/L	PMS 0.75 mM	TC	RR↓ from 0.025 mM (~ 98%) to 0.15 mM (~ 45%)	[102]
5–20 mg/L	Fe^0^@CNT 0.2 g/L	PMS 1 mM	SMT	RR↓ from 5 mg/L (99%) to 20 mg/L (~ 90%)	[88]
50–150 mg/L	Fe@C-PDA 0.2 g/L	PS 0.2 g/L	TC	RR↓ from 50 mg/L (99.7%) to 150 mg/L (92.7%)	[52]
5–60 μM	Fe_2_O_3_@CNT 0.015 g/L	H_2_O_2_ 50 mM	MB	RR↓ from 5 uM (100%) to 60 uM (~ 30%)	[56]
20–80 mg/L	NiCo@NCNT 0.05 g/L	PMS 0.65 mM	IBP	RR↓ from 20 mg/L (99%) to 80 mg/L (~ 75%) after third cycles	[55]
10–70 mg/L	Fe^0^/Fe_3_C@NC 0.2 g/L	PS 0.5 g/L	BPA	RR↓ from 10 mg/L (0.097 min^−1^) to 70 mg/L (0.006 min^−1^)	[134]
10–40 mg/L	Fe–N@C 40 mg/L	PMS 40 mg/L	OII	RR↓ from 10 mg/L (99%) to 40 mg/L (~ 50%)	[67]

Recently, PAA has garnered significant attention in water treatment applications due to its ease of use, strong sterilization capacity, and minimal production of hazardous byproducts [130]. Despite its potential, the activation of PAA using chainmail catalytic systems remains relatively underexplored. Xiao et al. [131] demonstrated that Cu@G could activate PAA to achieve 92.52% removal of SMT within 45 min, while H_2_O_2_ alone showed negligible degradation, highlighting its limited contribution to overall performance. More importantly, a recent study revealed that Co@CNT-activated PAA outperformed conventional oxidants, including PMS, PS, and H_2_O_2_, in the degradation of BPA [28]. The enhanced reactivity of PAA is primarily attributed to its asymmetric molecular configuration and relatively low O–O bond dissociation energy (E_O–O, PAA_ = 159 kJ mol^−1^), which is significantly lower than that of PMS (E_O–O, PMS_ = 317 kJ mol^−1^) [125, 130]. These findings suggest that the effective activation of PAA by carbon-encapsulated catalysts represents a promising direction for advancing chainmail catalysis in water treatment applications.

### Effect of reactant and catalyst dosages and pollutant load

High concentrations of micropollutants can significantly impede their degradation in chainmail catalysis systems. Generally, an inverse relationship is observed between initial micropollutant concentration and removal efficiency (Table 4). For example, in the Co@NCNT/PMS system, Orange II removal efficiency decreased significantly from 100 to 50% as the initial concentration increased from 10 to 40 mg/L [37]. This concentration-dependent reduction in performance has been consistently reported across a range of micropollutants and chainmail catalysts (Table 4) [56, 67, 132]. The decline is primarily attributed to the accumulation of refractory intermediates, which can deactivate active sites or compete with the parent compounds for ROS, ultimately reducing catalytic efficiency [10, 133].

Generally, increasing the dosage of both catalysts and oxidants enhances degradation performance in chainmail catalytic systems (Table 4). For example, Duan et al. reported a strong positive correlation between 4-CP degradation and the dosage of FeMn@NCNT (R^2^ = 0.98) and PMS (R^2^ = 0.81) [30]. This enhancement is expected, as higher dosages supply more active sites and facilitate greater ROS generation, thereby accelerating pollutant degradation [61, 130]. However, excessive loading of either component can have detrimental effects, a phenomenon observed in several studies [38, 48, 55, 116, 134]. At high concentrations, catalyst particles may aggregate, reducing their effective surface area and consequently lowering catalytic activity [48]. Similarly, surplus oxidants or radicals can trigger quenching reactions, which lower the availability of reactive species for pollutant oxidation [22, 34, 135]. Therefore, careful optimization of both catalyst and oxidant dosages is essential to maximize degradation efficiency while maintaining cost-effectiveness.

### Effects of pH, temperature, inorganic ions, and natural organic matter

Although carbon-encapsulated catalysts generally offer more stable performance across a broad pH range compared to their unconfined counterparts [25, 28, 56, 65], no consistent trend has emerged regarding the effect of solution pH on degradation performance in chainmail catalysis (Table 5). For example, the highest removal of TC using CuFe_2_O_4_@C/PDS was observed at pH 4 [34], whereas other studies have reported improved performance under near-neutral conditions [38, 56]. These discrepancies highlight the complex role of pH, which not only affects the dominant species of oxidants and micropollutants but also alters the surface charge and physicochemical properties of the catalyst itself [20, 22]. In one study, enhanced SMT removal by Cu@C/PAA at pH 7–9 (compared to pH 3–5) was attributed to synergistic effects, including increased adsorption capacity, more efficient PAA activation, and the higher reactivity of the deprotonated SMT species (SMT^−^) prevalent near neutral pH [131].

**Table 5:** Impact of water matrices on the removal performance upon chainmail catalysis

Operation conditions	Materials	Reactants	Micropollutants	Observations	Reference
*Water temperature*					
25–45 ℃	FeMn@NC 0.2 g/L	PMS 0.5 mM	SMZ 10 mg/L	RR↑ from 25 ℃ (0.116) to 45 ℃ (0.121 min^−1^)	[53]
15–30 ℃	Co@MOC 0.16 g/L	PMS 1.3 mM	TC 20 mg/L	RR↑ from 15 to 30 ℃	[116]
25–40 ℃	Co@NCNT 0.05 g/L	PMS 0.4 g/L	TC 30 mg/L	RR↑ from 25 ℃ (95%) to 40 ℃ (~ 100%)	[114]
15–35 ℃	Co–N@NC 0.1 g/L	PMS 0.1 g/L	SMX 20 mg/L	RR↑ from 15 oC (~ 80%) to 35 ℃ (~ 97%)	[54]
30–50 ℃	Fe/Fe_3_C@NCNTs 0.6 g/L	H_2_O_2_ 9 mM	NOR 0.063 mM	RR↑ from 30 to 50 ℃	[105]
25–45 ℃	Co@NC 0.025 g/L	PMS 0.75 mM	TC 0.05 mM	RR↑ from 25 to 45 ℃	[102]
10–40 ℃	Fe_3_O_4_@CNT 0.2 g/L	PS 0.5 mM	TL 8 mg/L	RR↑ from 10 ℃ (0.074) to 40 ℃ (0.135 min^−1^)	[48]
20–40 ℃	Co_3_O_4_@CNT 0.12 g/L	PMS 0.5 mM	NX 0.03 mM	RR↑ from 20 ℃ (0.043) to 40 ℃ (0.126 min^−1^)	[61]
5–40 ℃	Fe_2_O_3_@CNT 0.015 g/L	H_2_O_2_ 50 mM	MB 0.01 mM	RR↑ from 5 ℃ (60%) to 40 ℃ (~ 100%)	[56]
15–35 ℃	Fe_3_C@NCNT 0.2 g/L	PMS 0.2 g/L	Phenol 20 mg/L	RR↑ from 15 to 35 ℃	[103]
*pH*					
pH 2–10	CuFe_2_O_4_@PDA 0.2 g/L	H_2_O_2_ 0.25 M	MB 10 uM	RR inhibition follows order of pH 2 (75%) > pH 3 (80%) > pH 4–10 (90%)	[40]
pH 3–11	Fe@C-BN 20 mg/L	PMS 0.1 g/L	OII 20 mg/L	RR inhibition follows order of pH 3 ~ 6 > pH 8.8 > pH 9.5 > pH 11	[113]
pH 5–10	Fe^0^/Fe_3_C@NC 0.2 g/L	PS 0.5 g/L	BPA 50 mg/L	RR inhibition follows order of pH 5 (0.006) > pH 10 (0.008) > pH 7 (0.010 min^−1^)	[134]
pH 5–10	FeMn@NCNT 0.1 g/L	PMS 6.5 mM	Acetamiprid 7.8 μM	RR inhibition follow order of pH 10 (0.026 min^−1^) > pH 8 (0.059) > pH 6.5 (0.062) > pH 5 (0.064 min^−1^)	[39]
pH 2–10	Fe_3_C@NCNT 0.1 g/L	PS 1 mM	SMX 5 mg/L	RR inhibition follows order of pH 2 (0.051 min^−1^) > pH 8 ~ 10 (0.075) > pH 5 (0.084) > pH 7 (0.087 min^−1^)	[38]
pH 5–9	Fe@C-PDA 0.2 g/L	PS 0.2 g/L	TC 100 mg/L	No significant effects of pH (drop ~ 10%)	[52]
pH 3–9	MnFe_2_O_4_@C-NH2 1 g/L	H_2_O_2_ 29.4 mM	OFX 30 mg/L	RR inhibition follows order of pH 9 (54.8%) > pH 7 (66.4%) > pH 5 (72.5%) > pH 3 (97.4%)	[51]
pH 6–11	Fe_0_@C 0.25 g/L	PS 1 mM	4-CP 0.15 mM	RR inhibition follows order of pH 11 (20%) > pH 6 ~ 9 (90%)	[63]
pH 3–9	Fe_3_O_4_@CNT 0.2 g/L	PS 0.5 mM	TL 8 mg/L	RR inhibition follows order of pH 3 (50%) > pH 9 (60%) > pH 7 (89%)	[48]
pH 3–9	Ni@NC 0.1 g/L	PS 0.1 g/L	TC 20 mg/L	No significant effects of pH (drop ~ 10%)	[132]
pH 3–11	Co@NC 0.025 g/L	PMS 0.75 mM	TC 0.05 mM	RR inhibition follows order of pH 3 ~ 5 (~ 60%) > pH 7 (85%) > pH 9 ~ 11 (90%)	[102]
pH 3.3–7.8	Fe/Fe_3_C@NCNT 0.6 g/L	H_2_O_2_ 9 mM	NOR 0.063 mM	RR inhibition follows order of pH 7.8 (75%) > pH 4.6 ~ 6.2 (90%) > pH 3 (99%)	[105]
pH 3 -11	FeMn@NC 0.2 g/L	PMS 0.5 mM	SMZ 10 mg/L	RR inhibition follows order of pH 3 (0.049 min^−1^) > pH 11 (0.056) > pH 6 (0.111 min^−1^)	[53]
*Anion types and concentration*					
Cl, NO_3_, HCO_3_, HPO_4_	CuFe_2_O_4_@C 0.4 g/L	PS 1 mM	TC 50 mg/L	RR inhibition follows order HPO_4_ > HCO_3_ > Cl ~ NO_3_	[34]
0–50 mM				RR decreases with an increase in anion concentration	
HCO_3_, NO_3_, CO_3_, Cl, SO_4_	Co@NCNTs 5.08 mg/L	PMS	AO7 15 mg/L	RR inhibition follows order HCO_3_ > CO_3_ > NO_3_ ~ Cl ~ SO_4_	[84]
20 mM					
Cl, HCO_3_, H_2_PO_4_	FeMn@NC 0.2 g/L	PMS 0.5 mM	SMZ 10 mg/L	RR inhibition follows order H_2_PO_4_ > HCO_3_ > Cl	[53]
1–5 mM				RR decreases with an increase in anion concentration	
CL, HCO_3_, H_2_PO_4_, NO_3_, SO_4_	Fe_3_C/Fe@NC 0.3 g/L	PMS 0.1 mM	SMX 20 mg/L	RR inhibition follows order Cl ~ HCO_3_ > H_2_PO_4_ > NO_3_ ~ SO_4_	[97]
1 Mm					
HCO_3_, H_2_PO_4_, Cl, SO_4_, NO_3_	Co@MOC 0.16 g/L	PMS 1.3 mM	TC 20 mg/L	RR inhibition order of HCO_3_ > H_2_PO_4_ ~ Cl > SO_4_ > NO_3_	[116]
5–15 mM				RR decreases with an increase in anion concentration	
Cl, PO_4_, HCO_3_	Cu@G 0.02 g/L	PAA 0.2 mM	SMT 0.01 mM	RR inhibition follows order of HCO_3_ > Cl ~ PO_4_	[131]
0.5–2 mM				RR slightly decreases with an increase in anions	
Cl, HPO_4_, HCO_3_	Co–N@NC 0.1 g/L	PMS 0.1 g/L	SMX 20 mg/L	RR inhibition follows order Cl ~ HPO_4_ > HCO_3_	[54]
5 mM					
H_2_PO_4_, HCO_3_, Cl	FeMn@NCNT 0.1 g/L	PMS 1 g/L	4-CP 0.05 mM	RR inhibition follows order of H_2_PO_4_ ~ HCO_3_ > Cl	[30]
1–10 mM				No significant effects on RR with an increase anion concentration	
H_2_PO_4_, HCO_3_, Cl, NO_3_	Co@NC 0.025 g/L	PMS 0.75 mM	TC 0.05 mM	RR inhibition follows order of H_2_PO_4_ ~ HCO_3_ ~ Cl > NO_3_	[102]
5–10 mM				No significant effects on RR (~ 10%) with an increase in anion concentration	
CO_3_, NO_3_, Cl, H_2_PO_4_	Ni@NC 0.1 g/L	PS 0.1 g/L	TC 20 mg/L	RR inhibition follows order of CO_3_ > NO_3_ ~ Cl > H_2_PO_4_	[132]
0–10 mM				RR decreases with an increase in anion concentration	
*DOM types and concentration*					
1–5 mg/L HA	Cu@G 20 mg/L	PAA 0.2 mM	SMT 0.01 mM	RR↓significantly from 0 mg/L (92.5%) to 5 mg/L (~ 30%)	[131]
Ground water, River water				RR↓ slightly simulate ground water (80%) > actual groundwater ~ river water (~ 85%)	
	FeMn@NC 0.2 g/L	PMS 0.5 mM	SMZ 10 mg/L		[53]
Xianjiang River, Hou Lake, Tap Water, HA				RR inhibition follows order Xiangjiang river (~ 60%) > Hou lake (~ 65%) > Tapwater ~ HA (~ 80%)	
5–15 mg/L HA	Co@NCNT 0.05 g/L	PMS 0.4 g/L	TC 30 mg/L	RR↓ from 0 mg/L (90%) to 15 mg/L (~ 75%)	[114]
Lake water, Tap water				RR↓ slightly with lake water and tap water (< 10%)	
0–10 mg/L NOM	Co_3_O_4_@CNT 0.1 g/L	PMS 0.2 mM	PBA 0.1 mM	No significant effects of HA concentration on RR (drop ~ 10%)	[28]
Wastewater, Lake, Tapwater				RR↓ slightly with lake water and municipal wastewater	
1–8 mg/L HA	FeMn@NCNT 0.1 g/L	PMS 1 g/L	4-CP 0.05 mM	No significant effects of HA concentration on RR (drop < 5%)	[30]
10–20 mg/L HA	Co@NC 0.025 g/L	PMS 0.75 mM	TC 0.05 mM	RR↓ from 10 mg/L (> 80%) to 20 mg/L (~ 75%)	[102]
Tap water, river water				RR↓ slightly (~ 10%) with tap water and natural water	
0–10 mg/L HA	Ni@NC 0.1 g/L	PS 0.1 g/L	TC 20 mg/L	RR↓ from 0 mg/L (96.1%) to 10 mg/L (~ 20%)	[132]
Tap water, river water				RR inhibition follows order of natural water (~ 65%) > tap water (~ 80%)	
0–10 mg/L HA	Fe_3_O_4_@CNT 0.4 g/L	PS 0.5 mM	TL 8 mg/L	No significant effects of HA concentration on RR (drop < 10%)	[48]
Yangtze River, Xuanwu Lake				RR inhibition follows order of Xuanwu Lake (> 80%) > Yangtze River (~ 95%)	

It is well established that elevated temperatures enhance degradation efficiency in chainmail catalysis (Table 5). For example, the removal of Orange II by Fe@C-BN/PMS increased significantly from approximately 40% to 95% as the temperature increased from 10 to 55 °C [113]. A similar temperature-dependent improvement was reported in the Co_3_O_4_@CNT/PMS system, where the degradation rate of NOX tripled as the temperature increased from 20 to 40 °C [61]. This temperature effect is generally attributed to two primary mechanisms. First, the activation of oxidants through O–O bond cleavage is an endothermic process, meaning that higher temperatures lower the energy barrier for bond dissociation [107]. Second, elevated temperature improves mass transfer, resulting in more frequent collisions between reactants and catalysts [48]. Together, these effects enhance ROS generation and ultimately accelerate pollutant degradation.

Inorganic anions such as chloride (Cl‾), nitrate (NO_3_‾), carbonate (CO_3_^2^‾), bicarbonate (HCO_3_^2^‾), and phosphate (PO_4_^3^‾) are commonly found in natural waters, making their interaction with ROS unavoidable. In general, these anions negatively affect catalytic performance by competing with target pollutants for adsorption sites or scavenging ROS, particularly highly reactive species like •OH•, SO_4_•‾, CH_3_C(O)OO•, resulting in the formation of secondary ROS (e.g., Cl•, Br•, CO_3_•‾, NO_3_•‾) that exhibit lower redox potentials [17, 20, 22]. For example, in Fe_3_O_4_@CNT/PDS systems, the addition of 0, 2, 5, and 10 mM NaHCO_3_ led to a progressive decline in the degradation rate constant of TC, decreasing from 0.096 to 0.043 min^−1^ [48]. Similarly, a comparative study on the impact of various anions in FeMn@NC/PMS systems revealed that H2PO4- exerted the strongest inhibitory effect on SMZ removal (70%), followed by HCO3‾ (80%) and Cl‾ (95%), as compared to the controlled experiments (97%) at a similar concentration of 5 mM [53]. In another investigation, the extent of anion-induced inhibition on TC degradation using CuFe2O4@C/PS followed the order HPO42‾ (50%) > HCO3‾ (80%) ≈ NO3‾ (80%) > Cl‾ (~ 85%) at a concentration of 10 mM. When the concentration increased to 50 mM, HPO42‾ and HCO3‾ caused even stronger suppression of catalytic performance, whereas NO3‾ and Cl‾ showed little additional inhibitory effect, suggesting their saturated impact [34]. These findings demonstrate that both anion type and concentration could play a critical role in catalytic performance. In surface waters, anion levels can range up to several mM depending on watershed conditions, seasonal fluctuations, and agricultural inputs. Therefore, the background matrix of each water source must be carefully considered to ensure effective removal of micropollutants by chainmail catalysis.

DOM, like inorganic anions, is an essential component of natural aquatic environments. Comprising a heterogeneous mixture of organic compounds with diverse physicochemical characteristics, DOM originates from both biogeochemical processes and anthropogenic inputs [136]. Its concentrations can vary substantially from 5 to 30 mg C/L in aquatic systems [137], often three orders of magnitude higher than those of micropollutants. As a result, the influence of DOM on micropollutant degradation is inherently complex and concentration-dependent. At moderate levels, specific DOM constituents such as polyphenols and quinones can serve as electron shuttles, facilitating redox cycling of TMs like Fe(III)/Fe(II) [138], and Cu(II)/Cu(I) [139], thereby enhancing PMS activation and promoting efficient pollutant degradation. Conversely, at elevated concentrations, DOM is known to scavenge ROS, especially radical species, thereby suppressing catalytic activity [20]. In the context of chainmail-catalyzed AOPs, several studies suggest that the influence of DOM may be negligible [28, 30, 107]. For example, nearly complete degradation of AO7 was achieved within 30 min using Fe@NCNT/PMS, regardless of the DOM source present [107]. Similarly, Liu et al. [65] reported that increased DOM concentration ten times (from 0 to 10 mg/L) only led to a slight reduction in removal efficiency (< 10%) and degradation kinetics mostly remained unchanged irrespective of water resources, i.e., tap water, lake water, and municipal wastewater. More importantly, DOM exhibited a weaker inhibitory effect in Co@CNT/PAA systems compared to Co/CNT/PAA and Co3O4/PAA counterparts during BPA degradation. This reduced impact is likely due to a combination of synergistic factors: the selective action of non-radical species such as ^1^O_2_ in the presence of DOM, the protective size-exclusion properties of the carbon shell, and confinement effects associated with core–shell structures [28]. Despite these promising results, such results remain largely conceptual, as the practical application of cobalt-based nanocatalysts encapsulated in commercial CNTs is not yet viable for large-scale water treatment. Moreover, given the prevalence, high concentrations, and structural complexity of DOM in real water matrices, further studies are essential to elucidate its mechanistic interactions with micropollutants and chainmail catalysts. Critical questions remain unanswered: Which specific DOM fractions most significantly affect catalytic efficiency? How does DOM compete with micropollutants, whether for ROS or access to catalytically active sites? And what degradation pathways dominate in the presence of DOM? Addressing these questions is crucial to bridging the gap between controlled laboratory studies and the practical application of chainmail catalysis in real-world water treatment scenarios.

## Conclusions and future prospects

Amid growing concerns over the widespread occurrence of micropollutants in global water systems, the need for innovative water treatment technologies has become increasingly urgent. Recent progress in chainmail catalysis presents a compelling solution to address the limitations of conventional advanced oxidation processes (AOPs), which often suffer from decreased removal efficiencies over time. This review has highlighted the advantages of carbon-encapsulated catalysts and elucidated the underlying mechanisms underpinning their enhanced stability and degradation performance. The remarkable effectiveness of chainmail catalysts stems from the synergistic interplay between the robust outer carbon shell and the transition metal (TM) core, which together foster beneficial interfacial interactions. These structural features critically influence key parameters, including thermodynamic favorability, mass transfer dynamics, reactive oxygen species (ROS) generation, and pollutant degradation pathways. Furthermore, non-radical oxidation pathways, particularly those involving singlet oxygen (^1^O_2_) and electron transfer mediation (ETM), have emerged as important contributors to the overall catalytic efficacy.

Structural factors, including pyrolysis temperature, carbon shell morphology, and the metallic composition of the core, are pivotal in determining catalytic degradation performance. Extensive research has highlighted the importance of pyrolysis conditions and nitrogen doping in optimizing catalyst activity, while also emphasizing that ultrathin carbon layers and smaller particle diameters do not necessarily correlate with improved reactivity. In addition to structural design, operational parameters, including the type and concentration of oxidants, initial concentrations of pollutants and catalysts, as well as the composition of the water matrix, significantly affect treatment efficacy. Current literature indicates that the reactivity of oxidants generally follows the order of PAA > PMS > PS > H_2_O_2_, a trend largely attributed to differences in molecular asymmetry and O–O bond dissociation energy. Notably, the robust performance of chainmail catalysts across diverse water matrices further highlights their strong potential for real-world applications in micropollutant remediation.

Despite its potential, chainmail catalysis remains largely confined to fundamental research, currently aligning with a technology readiness level (TRL) of 4–6. Advancing this technology to the pre-pilot (TRL 7) and full pilot or demonstration stages (TRL 8–9) will require substantial experimental validation and scale-up efforts. To support this transition, several critical research questions must be addressed. A more comprehensive understanding of degradation mechanisms is essential, particularly regarding how chainmail catalysts modulate the generation and transformation of various ROS. For example, in 1D carbon nanotube (CNT)-based systems, it remains unclear whether ROS are preferentially generated at the external surface or within the inner cavity, and whether capillary effects facilitate enhanced reactant transport. The specific roles of ETM and metal–oxo species also warrant further investigation, particularly whether these species act directly in pollutant degradation or indirectly through the formation of intermediate oxidants. Additionally, a deeper theoretical framework is needed to clarify the thermodynamic drivers and mass transfer behaviors unique to chainmail systems. Key questions include the determinants of mass transfer rates, the mechanisms by which ROS migrate from catalyst surfaces, and the extent to which such processes occur at the nanoscale, especially in comparison to conventional AOPs.

For chainmail catalysis to be translated into practical water treatment applications, fabrication processes must be cost-effective and scalable. Future studies should focus on scalable and controllable synthesis routes for ultrathin carbon shells, particularly those ensuring nano- or even angstrom-scale precision and uniform interfacial bonding, which is essential for large-scale implementation. This calls for the continued development of TM nanocatalysts and organic precursors specifically optimized for industrial scalability. In parallel, a more systematic evaluation of oxidant systems is required. Although PAA exhibits higher reactivity compared to PMS and PS, its inherent instability and liquid-phase form present logistical challenges in terms of operational safety, storage, and transport. Additionally, the disparity between controlled laboratory experiments and the complexity of natural water matrices may obscure the true influence of operational parameters and matrix components. Systematic evaluation of long-term operation stability under continuous mode and real water matrices is also needed to evaluate catalyst resilience beyond lab-scale conditions. For example, it remains essential to determine which secondary reactive species emerge from the interactions between inorganic anions and primary ROS, whether these intermediates significantly impact removal efficiency, and if they contribute to the formation of toxic by-products. These considerations are particularly relevant in the treatment of seawater, saline water, and wastewater effluents.

Given the heterogeneity of DOM, further research is also necessary to elucidate how its concentration and chemical composition affect chainmail catalytic degradation. Identifying the specific fractions of DOM that exert the greatest influence, clarifying their competitive interactions with micropollutants for ROS or catalytically active sites, and mapping the resulting degradation pathways are critical steps toward closing the existing knowledge gap.

Finally, comprehensive techno-economic and environmental assessments of various oxidant systems are imperative to identify viable options for real-world implementation. Techno-economic analysis comparing chainmail catalysis with conventional AOPs should also be taken into consideration for identifying performance, capital, and operational costs that enable industrial translation. Addressing these challenges through in-depth investigation will be vital to bridge the current knowledge gap and enable the successful deployment of chainmail catalysis in real-world water treatment practices.

## Data Availability

The datasets used and/or analysed during the current study are available from the corresponding author on reasonable request.
